# Challenges in strengthening multi-sectoral action for optimum preparedness and response for public health emergencies in Sri Lanka

**DOI:** 10.1371/journal.pgph.0005964

**Published:** 2026-07-07

**Authors:** Suriyakumar Mahendra Arnold, Pasyodun Koralage Buddhika Mahesh, Dakshila Indikadahena Galappatti, Asela Gunawardena

**Affiliations:** Department of Health, Ministry of Health, Colombo, Sri Lanka; McGill University, MALAYSIA

## Abstract

Sri Lanka is a lower-middle-income island-nation that often contends with multiple public health emergencies (PHEs) such as floods and disease epidemics. In preparing and responding to these PHEs, different units of the Ministry of Health (MOH) take responsibility for the functions of a typical National Public Health Agency in Sri Lanka. This study was done to describe current approaches within multisectoral action and to explore the challenges to multisectoral action involving the MOH in preparedness for and response to PHEs. The study included a risk assessment, stakeholder-mapping, a desk- review of literature and documents available with stakeholder institutions. Additionally, we conducted qualitative interviews with key informants relevant to PHE response covering nine provinces. The risk assessment was done to identify potential PHEs followed by stakeholder mapping. The desk review aimed to identify the deficiencies of the existing major policy documents for multisectoral action. The study team conducted 51 KIIs from April to May 2025 to synthesise challenges using a semi-structured questionnaire with purposively selected team members involved in different aspects of multisectoral action. The US CDC model on public health linkages served as the conceptual framework, including four domains: 1) policies/agreements/legislation, 2) infrastructure, 3) governance/funding/logistics, and 4) people-organization culture. Thematic coding analysis of qualitative data was performed. The main challenges imposed by public policy weaknesses related to multisectoral action included: lack of inclusive engagement of stakeholders in policy formulation, not having a strong policy architecture for collaboration, non-adaptive policies and vague terminology. Challenges imposed by policy-deficiencies related to other three domains included: sub-optimum data management, delayed fund mobilization, lack of mediation of activities of NGOs, less-customized risk communication, and ad-hoc collaborative efforts between stakeholders. The study recommends adapting existing policy documents, clarifying decentralization and sub-national implementation arrangements, establishing inter-stakeholder operational guidelines, and strengthening intersectoral awareness of roles and communication mechanisms.

## Introduction

Public Health Emergencies (PHEs) encompass acute events – natural or man-made – that challenge health systems due to their scale, timing and unpredictability overwhelming the system capacity [1,2]. The COVID-19 pandemic shed light on the importance of rapid and coordinated health system responses to PHEs [3,4]. PHEs threaten lives and disrupt economies. It is therefore essential to effectively organize health systems to enhance their response capacity and resilience. PHEs pose direct and indirect threats to all building blocks of the health system- service delivery, health workforce, information systems, access to medicines, financing, and leadership/governance [2,5]. Multisectoral action is necessary to strengthen all these building blocks. This study examined approaches and challenges in multisectoral action for enhancing health system preparedness for PHEs, drawing on experiences in Sri Lanka.

Sri Lanka, a lower-middle income country with a population of approximately 22 million is no exception to the impact of PHEs with periodic events like floods, adverse weather conditions, and epidemics including dengue and COVID-19. The World Health Organization (WHO) states that multisectoral actions can strengthen “country ownership, accountability, stewardship of resources, and organizational effectiveness around health emergency preparedness, readiness and response” [6]. Further, when multisectoral action is taken during the preparatory phase rather than the response phase, the benefits are greater [6].

Many models and frameworks have been developed to depict the level of preparedness and response for PHEs through multisectoral action [5,7–12]. The multisectoral action in the form of preparedness coordination reflects “deliberate collaboration between stakeholders from multiple and diverse sectors and disciplines working towards the shared goal of enhanced health emergency preparedness by leveraging knowledge, expertise, strengths, reach and resources” [6]. US-CDC has proposed a comprehensive framework to highlight “information on practical approaches public health leaders may consider using to strengthen linkages and their enablers for improved public health impact in their own national contexts” [13]. These linkages have been defined as “practical, replicable activities or actions that facilitate collaboration between public health functions or organizations to improve public health impact” [13]. In this model, the linkages have been elaboratively categorized under five levels; functional, multifunctional, multisectoral, multilevel and international [13]. Four enablers have been mentioned in this model: “policies, agreements and legislation”, “infrastructure”, “governance, funding and logistics” and “people and organizational culture” [13].

While all these four enabler-domains are important, “policies, agreements and legislation” drive the coordination of multisectoral response in PHEs. Out-of-date policies and lack of understanding on the available policies have been identified as challenges to optimum response in PHEs in the global literature [2]. Meanwhile, characteristics of a successful health system include resilience and the capacity to learn from experience, feeding knowledge and data back into the policy [14].

Literature on “policies, agreements and legislation” for multisectoral action for PHEs in Sri Lanka is scarce and has not been consolidated. Thus, the current study aimed to describe the current risk of PHEs in Sri Lanka, and to examine the approaches and challenges of multisectoral action in response efforts, with particular emphasis on policies, agreements, and legislation.

## Methods

### Ethics statement

Ethics approval was obtained from the Ethics Review Committee of National Institute of Health Sciences (NIHS/ERC/24/77R).

The study included a national risk assessment for PHEs, stakeholder mapping, desk review, and a qualitative study covering respondents within the nine provinces of Sri Lanka [15,16].

The risk assessment was conducted to determine the priority PHE risks presently faced by Sri Lanka, which warrant multisectoral action. This was conducted with secondary data available at Disaster Preparedness and Response Division, Provincial Directorate of Health Services and Regional Directorate of Health Services for the last 10 years as well as with inputs from the experts in those institutions. The secondary data included preparedness plans for PHEs and reports available on response phases within the same institutions mentioned above. The actual frequency of events, likelihood of the event and the impact of the event were assessed [17]. In addition, the details about the stakeholders and their extent of involvement were asked from the experts in the above institutions.

A stakeholder mapping exercise was conducted to identify the major stakeholders of multisectoral action and assess their degree of engagement in PHEs. The stakeholders who have been involved or who are expected to be involved in potential events were also documented, following guidance from the WHO “Multisectoral preparedness coordination framework” [6]. The details obtained from the experts in the risk assessment was used in this process. Each stakeholder was rated as “high or low” for the two domains of “impact” and “influence” [18]; “impact” was defined as “how much of an impact does the stakeholder have on preparedness and response on PHEs” and “influence” was defined as “how much authority does the stakeholder have on preparedness and response on PHEs” [18]. Those with high levels of “impact” and “influence” were identified. Level of engagement was categorized as: consult (high interest- high influence), convince (high influence- low interest), update (high interest- low influence) and monitor (low influence- low interest) [18]. Co-authors discussed these assignments for consensus, and where needed, also consulted the technical experts in the institutions who are related to multisectoral actions. Participants for KIIs were recruited from the institutions identified as having “consult” and “convince” levels of engagement (Table 1) [18].

**Table 1:** KIIs conducted in different institutions.

Type of institution	Number of KIIs
Line Ministry health institutions (Disaster Preparedness and Response Division, Epidemiology Unit, Epidemiology Unit, National Dengue Control Unit, Anti-Malaria Unit)	05
Offices of Provincial and Regional Directors of Health Services (PDHS & RDHS)	28
Offices of Medical Officer of Health (MOH)	04
Curative health institutions (District General Hospital)	02
Disaster Management Centre	03
Provincial Council, District Secretariat, Divisional Secretariat	04
Security forces	02
Academia	01
NGOs	02
Total	51

A desk review was conducted to collate evidence for facilitators and barriers for multisectoral action and its governance. This included an online search in PubMed and Google Scholar and a review of reports on preparedness planning and responses from the various stakeholders. Expert feedback provided recommendations for additional grey literature and policy documents to include for review. In PubMed, subject heading and keyword searches were done. The subject heading search was done by exploring the relevant MeSH terms and keywords. The complete search strategy is shown in S1 Fig. Advance search option was used in Google Scholar. The reports that were reviewed included documents related to PHE preparedness and response activities available with stakeholders.

Interviews were conducted with stakeholders with experience working in the institutions that were determined to have both high impact and influence in multisectoral action [19–26]. Altogether, the team conducted 51 KIIs between April and May 2025 following recommended methodology [24–26]. Purposive sampling was conducted by discussing with staff members of selected institutions. Participants included different team members involved in: policy planning, implementation, research, administration, training, and financing. We reached out to each KI via the heads of their respective institutions. Furthermore, we ensured that the participants covered both government sector and non-governmental sectors, such as the Sri Lanka Red Cross. In the government sector, stakeholders from national, provincial, district and divisional levels were included. There are 9 Provincial Directorates of Health Services (PDHS offices) and 26 Regional Directorates of Health Services (RDHS offices) in Sri Lanka, hence more participants were from those institutions. Both curative and preventive sectors as well as health and non-health sectors were covered. The institutional breakdown of the 51 KIIs conducted is shown below in Table 1.

Representation of the Provincial/ District health staff in the KIIs is shown in S2 Fig.

A semi-structured questionnaire was prepared. The contents of the questionnaire were based on the four-domains of the US CDC framework (S1 Text). In this model, four enablers of public health linkages have been proposed as: 1). Policies, agreements and legislation, 2). Infrastructure, 3). Governance, funding and resources and 4). People and organizational culture. Interviews were done by study investigators with one being the moderator and another being a note taker. The average duration of a KII was 20 minutes (range 15–40 minutes). Interviews were audio-recorded with written consent given by participants. The audio recordings were transcribed following principles from Baily et al (2008) by two authors independently and spot-checked for quality. Data were analysed and interpreted concurrently using a convergent-parallel design to integrate findings across methods [27]. Thematic analysis was done using the deductive approach under the four domains of the US CDC framework [28].

## Results

The results are synthesized and presented using research conducted under the following main approaches: 1. Risk Assessment and stakeholder mapping 2. Desk review of reports 3. Key Informant Interviews on 51 participants.

### PHEs (encountered and potential) of Sri Lanka and stakeholder-grid

The risk assessment revealed that PHEs in the previous 10 years included: COVID-19, dengue outbreaks, floods, landslides, explosions, and events due to adverse weather conditions. In addition, potential future event risks include: tsunami, other disease epidemics, chemical accidents, and recurrences of those that have been reported within last 10 years. The stakeholder-grid that was prepared to identify the organizations from which to draw the KII respondents is shown below in Table 2.

**Table 2:** Stakeholder grid.

Organization	Type of influence	Level of impact	Level of influence	Level of engagement
Disaster Management Centre	-Policy formulation -Administration -Coordination	High	High	Consult
Disaster Preparedness and Response Unit- Line Ministry	-Policy formulation -Coordination	High	High	Consult
Offices of Provincial Director of Health Services	-Implementation -Technical	High	High	Consult
Offices of Regional Director of Health Services	-Implementation -Technical	High	High	Consult
Epidemiology Unit	-Policy formulation -Technical	High	High	Consult
Family Health Bureau	-Policy formulation -Technical	High	High	Consult
Health Promotion Bureau	-Policy formulation -Technical	High	High	Consult
Environmental & Occupational Health Unit	-Policy formulation -Technical	High	High	Consult
National Dengue Control Unit	-Policy formulation -Technical	High	High	Consult
Anti Malaria Campaign	-Policy formulation -Technical	High	High	Consult
Provincial Councils	-Administration -Implementation -Financial	High	High	Consult
District Secretariate	-Administration -Implementation -Financial	High	High	Consult
Divisional Secretariate	-Administration -Implementation	High	High	Consult
Offices of Medical Officers of Health	-Implementation -Technical	High	High	Consult
Curative institutions	-Implementation -Supportive	High	High	Consult
Security forces	-Supportive -Implementation	High	High	Consult
Non-Governmental Organizations	-Supportive -Financial	High	Low	Convince
Universities	-Technical	High	Low	Convince

Except for the NGOs and universities, all other institutions mentioned in the above table were categorized as having a high level of impact and influence and thus being in the “consult” level of engagement. The former two were assigned the “convince” level of engagement [18].

### Policy documents

From the desk review and the discussions with experts in the institutions, eight policy documents related to multisectoral action were identified. These eight policy documents were categorized as having: 1) general components of disasters, 2) health-related disasters as the main focus, 3) risk communication, and 4) security as the main focus. An analysis of the policy documents related to multisectoral actions in PHEs and their gaps are shown in Table 3 with a summary of each provided below.

**Table 3:** Existing policy documents and their gaps.

Policy document	Deficiencies
**Policies related to general components of disasters as the main focus**	
1. Disaster Management Act No. 13 of 2005	Designed mainly for natural disasters and not for disease epidemics and pandemics. Coordination on preparedness and response of DMC based on disease surveillance is not optimal with health and animal sector.
2. National Disaster Management Plan, 2023–2030	No policy mandate for real time integrated Disease Surveillance System available, which hinders early risk mitigation and prevent health emergencies.
3. National Emergency Operation Plan (NEOP)	IHR requires detailed capacities in surveillance and laboratory diagnostics. The NEOP lacks explicit references to national laboratory networks or protocols for epidemiological surveillance.
**Policies related to disasters with health as the main focus**	
4. National Health Policy 2016 – 2025	Institutionalization of multisector coordination is weak in the policy text. While the policy endorses intersectoral action, it stops short of prescribing statutory or permanent governance arrangements such as having district multisector fora with defined mandates and reporting. Subnational operational capacity and decentralisation not fully detailed - the Policy’s national orientation needs clearer provisions for district/divisional roles and resourcing.
5. National Health Strategic Master Plan 2016 – 2025	Human Resources for Health (HRH) strategies exist but implementation gaps like uneven distribution, retention problems in rural/estate areas, unclear task-shifting policies persist. Strengthening HRH was repeatedly identified as a constraint. The plan references eHealth but interoperability and nationwide data governance were not fully implemented. This created fragmented records and poor information flows. The Master Plan recognises the private sector but lacks a strong, operational regulatory framework and clear engagement models to align private provision with public goals.
**Policies related to risk communication**	
6. National Risk Communication Strategic Plan for Public Health Emergencies in Sri Lanka	Although the plan emphasizes community engagement, there is little indication of tailored messaging for different socio-cultural groups like urban slum dwellers and estate communities beyond general language translation.
**Policies related to security**	
7. Public Security Ordinance	There is no dedicated mechanism to ensure routine collaboration between the Ministry of Health and the Ministry of Defense for health emergency readiness.
8. Sri Lanka Police Ordinance	The Police Ordinance does not explicitly include public health as an area of responsibility, leading to potential legal uncertainties when police are called upon for health-related duties.

The Disaster Management Act gave the opportunity to form the Disaster Management Centre (DMC) which is the lead agency for coordinating preparedness, mitigation, and recovery efforts in emergencies/disasters [29]. The National Disaster Management Plan (NDMP) 2023–2030 serves as Sri Lanka’s comprehensive framework for disaster risk management [30]. It encompasses various hazards, including health emergencies such as epidemics and pandemics. The National Emergency Operation Plan (NEOP), provides a comprehensive framework for coordinating emergency responses across various hazards aiming to enhance national resilience through multi-sectoral coordination, early warning systems, and standardized response protocols [31]. The Sri Lanka National Health Policy (2016–2025) is built upon the concept of “Universal Health Coverage” (UHC), aiming to ensure accessibility to quality services with financial protection. The National Health Strategic Master Plan 2016–2025 underscores a commitment to addressing emerging health challenges, improving infrastructure, and ensuring compliance with international health standards, including the IHR 2005 [32]. The National Risk Communication Strategic Plan of Sri Lanka aims to strengthen Sri Lanka’s capacity to provide accurate information, counter misinformation, and build public trust in health interventions. The Public Security Ordinance No. 25 of 1947 is a key legal instrument in Sri Lanka that enables the integration of public health and security authorities during emergencies, including pandemics and disease outbreaks [33]. Although the primary mandate of Sri Lanka Police Ordinance No. 16 of 1865 is on law enforcement and community policing, its provisions offer potential leverage points for extending the role of the police force in PHEs [34].

### Current structure of delivery of NPHA functions in Sri Lanka

Following the desk review and discussions with the institutions, the current mechanisms of delivering NPHA functions were summarized. Currently, there is no single entity named as a National Public Health Agency (NPHA) in Sri Lanka. The central Ministry of Health issues guidelines while the provincial, district, and divisional level stakeholders contribute to the implementation. At the central level, the Quarantine Unit and Epidemiology Unit function as co- national focal points under the International Health Regulations IHR 2005 while the latter leads in disease surveillance as well. Health sector preparedness and response in general are coordinated by the Disaster Preparedness and Response Division of Ministry of Health. There are other specific control programs like the National Dengue Control Unit and the National Tuberculosis and Chest Diseases Control Programme. The non-health stakeholders like the Provincial Councils, Local Government bodies, District Secretariates and Divisional Secretariates play critical coordination and response-implementation roles in the preparedness and response in PHEs.

Building on the limitations identified in the desk review, challenges to multisectoral action as suggested by the key informants are shown below in Table 4. The experts pointed out eight challenges in relation to policy-agreement-legislation domain, highlighting deficiencies in policy focus, priorities, architecture, implementation, and monitoring. The second domain that covered physical and digital infrastructure yielded seven challenges that covered aspects like lack of common physical infrastructure as well as sub-optimal data management. Challenges in relation to fund mobilization, donation processes, and NGO coordination were highlighted for the domain on “governance-funding and resources”. Six challenges that covered aspects like training fora, scopes of work, standard operating protocols, sustainability, and integration of social media were described under the domain on “people-organization culture”.

**Table 4:** Challenges as suggested by the Key Informants.

Policies- agreements-legislation domain	Infrastructure domain
• Major policy and legislative instruments emphasize response actions more than preparedness • Priorities are not aligned across sectors in relation to PHEs • No strong policy architecture is present for collaboration between actors • NGOs have to fulfil their own funders’ requirements • It is often necessary to go through default hierarchies of the administration in incident commanding system, limiting efficiency • There is a lack of documented policies on data sharing • There is a lack of monitoring mechanisms for multisectoral action for PHEs • There is a lack of policies on documentation in relation to preparedness and response for PHEs	• Limited identification of a common infrastructure for the response phase was noted • Non-availability of an integrated laboratory network was found • There is a lack of resource mapping across different sectors • Fragmented data gathering is observed leading to duplication of efforts • There is no integrated database • Discrepancies between data collected from different sources are noted • Minimum use of digital technology in data management is seen, leading to more manual efforts
**Governance, funding and resources domain**	**People and organization culture domain**
• Non-availability of a separate fund to mobilize in PHEs was noted • Undue delays in fund mobilization are experienced • There is no mechanism to jointly monitor the functions and processes related to PHEs • Delays in mobilizing and redistribution of resources in the response phase are noted • Duplication is found in activities done by NGOs in some instances • There is lack of clarity on donation processes	• No common training fora are found • Lack of awareness on others’ scope in PHEs was noted • Lack of clarity on SOPs on other stakeholders was noted • Coordination often depends on personal connections that may not sustain with changes in human resources • Non-health focal points sometimes lack awareness on and knowledge of health system operations and needs • Non-availability of a system to use the beneficial effects and to reduce harmful influence of social-media was found

By collating the evidence from the literature review, review of reports and KIIs, the main challenges in relation to policy deficiencies were synthesized. A key challenge identified was the lack of inclusive engagement of stakeholders in policy formulation, which has led to fragmented implementation. This challenge is further exacerbated by the absence of a strong policy architecture to support collaboration between actors and to enable timely adaptation to meet changing needs. As a result, outdated and uncoordinated policies create uncertainty about how provisions apply, particularly within a decentralized system, and contribute to inefficiencies in administrative, operational, and financial processes during PHEs. Another challenge identified is the absence of policy provisions for a common database, limiting the opportunities for data integration across sectors. The lack of an established laboratory network represents a manifestation of this gap. Additionally, vague terminology within security polices was highlighted as another challenge as it gives rise to uncertainties in interpretations.

## Discussion

This is the first documented study conducted in Sri Lanka at the national level on approaches and challenges for effective multisectoral action in PHEs. The findings revealed current PHE risks, identified key stakeholders for multisectoral action in PHEs in Sri Lanka, and explored potential challenges in relation to the four domains of the US CDC model: policies/ agreements/legislations, infrastructure, governance/funding/logistics, and people/ organizational culture. Our stakeholder mapping exercise identified a large number (n = 16) of categories of stakeholder institutions that were considered “high” in terms of their influence and impact, and in totality, represent a complex environment of stakeholders that require coordination and consultation. The findings suggest that there is no uniform framework that units are using for multisectoral governance. Many of these recommendations presented within the findings of this study also align with the UN Sendai Disaster Risk Reduction Framework [35].

Several methodological strengths which are potentially applicable in other settings too, can be noted. The stakeholders and their level of engagement were identified with the inputs of experts who actually involve in multisectoral activities within related institutions. KIIs were conducted covering all types of stakeholders with high influence and impact. The stakeholder engagement and the KIIs have strengthened inclusion of wide-range of actors across institutions. The synthesis of the challenges according to US CDC model ensured the comprehensiveness of findings as well as enabled their potential comparison with other settings.

Under “policies, agreements, and legislation”, the major challenges identified included policy deficiencies related to policy architecture, policy inclusiveness, policy adaptation, and vague policy terminology. These deficiencies are interconnected and contribute to the root cause of many other challenges in achieving multisectoral action for health. For example, non-adaptive and out-of-date policies hinder optimal functioning and efficient processes within decentralized governance structures. In addition, vague terminologies in legal policy documents, particularly around security-related policies, create uncertainties in interpretation and constrains their application in supporting PHE response efforts. Emphasizing the importance of clarifying legal provisions, the National Summit on Public Health Legal Preparedness in 2007 highlighted the need for ensuring legal preparedness in the context of PHEs [24]. Limited enforceability, duplication of efforts, fragmented involvement, vague mandates in inter-ministerial level are several policy-related challenges identified in global literature which are aligned with those elicited in the present study [36–40].The reported literature shows the deficiencies of the health policy processes hindering aspects like inclusiveness and meaningful engagement in PHEs [36–40].

The identified policy deficiencies lead to difficulties in relation to other domains of the US CDC framework as well. One example is fragmented coordination of the utilization of digital infrastructure (i.e., the infrastructure domain) leading to duplications in data management. For example, in a flood event, data are collected by the different units of the Ministry of Health itself using different data templates that creates unnecessary workloads for ground-level staff. This was again apparent in the response events related to Ditwah cyclone in late 2025 as well [41]. In epidemic events, identified deficiencies give rise to undue delays in funding mobilization that include transferring of funds along traditional hierarchical levels rather than having a more agile system in place as part of preparedness activities. In general, the absence of policy provisions to mandate and enable multisector collaboration and coordination, shared preparedness training platforms, and a common understanding of roles, scope of work, and resources across sectors, contributes to inefficiencies in response efforts. For example, health units may be unable to leverage support from non-health stakeholders during the response phase if roles and responsibilities are not clearly understood. Hence addressing the policy domain as a primary driver for multisectoral action, serves to intervene in challenges under other domains as well.

The importance of policy provisions is further highlighted by the absence of a provision to have a structural coordinating agency like an NPHA. Coordinating agencies are particularly critical as poor stakeholder engagement leads to fragmentation of services in relation to preparedness and response to PHEs(39). Even though the services related to PHEs are delivered from different units within the Ministry of Health, this fragmentation leads to duplication and gaps in some control measures. Similarly, the lack of coherent implementation and central monitoring lead to misalignment of priorities across organizations.

Under infrastructure, the major challenges identified in the study were non-identification of common physical infrastructure and sub-optimal data management. The absence of an integrated real-time database and lack of documented policies on data sharing creates a vicious cycle leading to poor data integration and management. This challenge was also identified in a mixed methods study in Ethiopia where limited digital infrastructure and incompatibility of data formats were identified as barriers to effective data coordination [40]. Sri Lanka should develop integrated unified databases to be used in PHEs to integrate operational data. Currently such databases – linking curative and preventive efforts – are found in relation to communicable conditions like dengue and tuberculosis. Once developed, such a platform can integrate health surveillance data with data from other sectors facilitating initiatives like the One Health approach [42]. The absence of a laboratory network, which was highlighted as another challenge, can also be addressed by a unified database and real-time data exchange. Thus, the main national laboratory should be linked with those at peripheral levels and this network could be incorporated to the integrated database with surveillance data.

For the “governance-funding and resources” domain, several financial difficulties were highlighted. Given this context, lack of an anticipatory pre-arranged financing mechanism makes the country more vulnerable [43]. Outdated financial procedures should be reviewed and policy adaptations undertaken. Under the domain of people and organization culture, it was elicited that most connections between organizations are on a personal level and not due to system-driven mechanisms, policies, or practices. In contrast, the better approach is to have well-laid system-level planning which is more sustainable [44]. A multi-site qualitative study has shown that, although ad-hoc interactions between professions may provide some benefit, it would not give rise to “systematic forms of collaboration” [45]. The involvement of NGOs yields potential benefits; however, ensuring non-duplication and balancing the commercial interests pose challenges. As an example, in the Ditwah response period, several NGOs were interested in arranging medical camps, yet the funders’ interests resulted in potential duplications. In some settings, the same geographical area was targeted due to funders’ interests. Balancing the commercial interests with health policies, like in the case of child milk-powder promotion, needed extra efforts.

The WHO has emphasized the value of proactive considerations and far-sighted policies over limited response measures [6]. Within this context, the way forward must include several considerations as shown in Table 5.

**Table 5:** Recommendations in addressing the challenges.

Core-challenges	Key recommendations
Absence of a strong policy architecture to support collaboration between actors and to enable timely adaptation to meet changing needs	Policy adaptations with timely revision of currently existing policy documents
Inefficiencies in administrative, operational, and financial processes during PHEs specially within the decentralization	Defining clearer terms in relation to decentralization and sub-national implementation
Lack of inclusive engagement of stakeholders in policy formulation and fragmented implementation	Better sectoral dialogues
Absence of policy provisions for a common database	Raising intersectoral awareness on mutual scopes
Vague terminology within security polices	Having specific inter-stakeholder operational guidelines

Policy adaptations with timely revision of currently existing policy documents are one such worthy endeavour. Within this, defining clearer terms in relation to decentralization and sub-national implementation are recommended. Having specific inter-stakeholder operational guidelines, raising intersectoral awareness on mutual scopes, and better sectoral dialogues are other key considerations. Once the policy domain is addressed, it will facilitate addressing the challenges of the other three domains as well. Challenges like the presence of data discrepancies, fragmented data gathering, absence of a common database can be solved once the data sharing provisions are thus streamlined through policies. At the same time, the funding related challenges mentioned under domain three can also be addressed and the human resource related challenges such as training and raising the awareness will also be facilitated once the policies are clearer with the policy reforms.

There were several limitations of the study. The challenges identified in the study were based on the subjective experiences and opinions of individuals and may be informed by one event or limited to one setting. To mitigate the potential for bias, a larger number of KIIs across a range of organizations – identified in the stakeholder mapping exercise – were conducted to ensure the inclusion of diverse perspectives and to strengthen the robustness of emergent themes.

## Conclusions and recommendations

This novel study identified critical challenges with multisectoral collaboration in Sri Lanka, involving policy, infrastructure, funding, and people organization culture. We found that services related to PHEs are delivered by different units in Sri Lanka without the existence of an NPHA entity to support coordination. Policy deficiencies give rise to challenges including lack of inclusive engagement of stakeholders in policy formulation leading to fragmented implementation, absence of a strong policy architecture for collaboration, lack of policy adaptation and vague terminology giving rise to uncertainties in interpretations. In addition, policy deficiencies impose challenges for the other three domains of the US CDC framework as well. These include: sub-optimal data management, delayed fund mobilization, suboptimal coordination of NGOs, and ad-hoc collaborative efforts between stakeholders. To address these challenges, policy adaptation of existing policy documents, defining clearer terms in relation to decentralization and sub-national implementation (i.e., clarifying roles, authority, and responsibilities), having specific inter-stakeholder operational guidelines, raising intersectoral awareness on mutual scopes and system-driven intersectoral communication are recommended. Following these recommendations can provide a foundation for a well-coordinated multisectoral approach in PHE preparedness and response in Sri Lanka.

## Supporting information

S1 Fig Search strategy. (DOCX)

S2 Fig Representation of the Provincial/District health staff. (DOCX)

S1 Text Semi structured questionnaire. (DOCX)
