# Supplementary material for: Challenges in strengthening multi-sectoral action for optimum preparedness and response for public health emergencies in Sri Lanka
Source: PLOS Glob Public Health. 2026 Jul 7;6(7):e0005964. doi: 10.1371/journal.pgph.0005964 (PMC13340774; doi:10.1371/journal.pgph.0005964)
Supplement: S1 Fig — (DOCX) [file pgph.0005964.s001.docx]

**S1_Fig: Search strategy**

| **Search number** | **Query** | **Results** |
| --- | --- | --- |
| 9 | ((((multi sectoral OR multi sector OR cross-sector OR intersectoral) OR (intersectoral collaboration[MeSH Terms])) OR (linkage*)) AND ((Public Health emergency OR NPHA OR national emergency OR disaster) OR (disaster management[MeSH Terms]))) AND (barriers OR challenges OR obstacles OR difficulties OR issues) | 2,245 |
| 8 | barriers OR challenges OR obstacles OR difficulties OR issues | 3,130,749 |
| 7 | (Public Health emergency OR NPHA OR national emergency OR disaster) OR (disaster management[MeSH Terms]) | 921,918 |
| 6 | disaster management[MeSH Terms] | 28,154 |
| 5 | Public Health emergency OR NPHA OR national emergency OR disaster | 921,918 |
| 4 | ((multi sectoral OR multi sector OR cross-sector OR intersectoral) OR (intersectoral collaboration[MeSH Terms])) OR (linkage*) | 207,639 |
| 3 | linkage* | 192,545 |
| 2 | intersectoral collaboration[MeSH Terms] | 2,865 |
| 1 | multisectoral OR multisector OR cross-sector OR intersectoral | 12,810 |
