# Supplementary material for: Challenges in strengthening multi-sectoral action for optimum preparedness and response for public health emergencies in Sri Lanka
Source: PLOS Glob Public Health. 2026 Jul 7;6(7):e0005964. doi: 10.1371/journal.pgph.0005964 (PMC13340774; doi:10.1371/journal.pgph.0005964)
Supplement: S2 Fig — (DOCX) [file pgph.0005964.s002.docx]

**S2_Fig: Representation of the Provincial/ District health staff**

| **Province** | **District** | **Number of KIIs** |
| --- | --- | --- |
| Central | Kandy  Matale  Nuwaraeliya | 3 |
| Eastern | Ampara  Batticaloa  Trincomalee | 3 |
| North Central | Anuradhapura  Polonnaruwa | 2 |
| Northern | Jaffna  Kilinochchi  Mannar  Mullaitive  Vavuniya | 3 |
| North Western | Kurunegala  Puttalam | 2 |
| Sabaragamuwa | Kegalle  Ratnapura | 2 |
| Southern | Galle  Matara  Hambantota | 3 |
| Uva | Badulla  Monaragala | 2 |
| Western | Colombo  Gampaha  Kalutara | 8 |
