# Supplementary material for: Challenges in strengthening multi-sectoral action for optimum preparedness and response for public health emergencies in Sri Lanka
Source: PLOS Glob Public Health. 2026 Jul 7;6(7):e0005964. doi: 10.1371/journal.pgph.0005964 (PMC13340774; doi:10.1371/journal.pgph.0005964)
Supplement: S1 Text — (DOCX) [file pgph.0005964.s003.docx]

**S1_Text: Semi structured questionnaire**

**Semi structured questionnaire**

Questions and prompts:

1. What kind of public health emergencies does your institution deal with?

Probe for:

- What have been already dealt with in the last ten years
- What can be anticipated in the coming ten years

2. What can you say about the preparedness of your institution for public health emergencies?

3. Can you explain how does your institution engage in multisectoral collaboration in preparedness and response to public health emergencies?

3. What challenges do you perceive in multisectoral collaboration and its governance in relation to policies, agreements and legislation

Probe for

- Legislative mandates
- Policies governing national– subnational relationships
- Rules and SOPs
- Data-sharing policies and MOUs

4. What challenges do you perceive in multisectoral collaboration and its governance in relation to infrastructure

Probe for:

- Physical structures for collaboration
- Public health observatories
- Information systems optimized for data sharing
- Conceptual or organizational frameworks for integration
- Regional public health assets

5. What challenges do you perceive in multisectoral collaboration and its governance in relation to governance, funding, and resources

Probe for:

- Political support for linkages
- Funding and resources to support subnational activities,
- Participation of subnational authorities in planning and implementation lifecycle
- Effective leadership and management of linkages
- Aligned or shared goals,
- Strategic plans,
- Work plans

6. What challenges do you perceive in multisectoral collaboration and its governance in relation to people and organizational culture

Probe for:

- Inclusivity of teams
- Trust
- Relationships and history of collaboration
- Effective internal communication
- Shared nomenclature
- Investing in skills and professional

7. What else have you got to say about preparing and responding to public health emergencies as far as your institution is concerned?

Thank you for your participation!
