# Supplementary material for: Challenges in strengthening multi-sectoral action for optimum preparedness and response for public health emergencies in Sri Lanka
Source: PLOS Glob Public Health. 2026 Jul 7;6(7):e0005964. doi: 10.1371/journal.pgph.0005964 (PMC13340774; doi:10.1371/journal.pgph.0005964)
Supplement: Attachment — Submitted filename: PGPH-D-26-00295_R1_SLB.pdf [file pgph.0005964.s005.pdf]

PLOS Global Public Health

Challenges in strengthening multi-sectoral action for optimum preparedness and response for public health emergencies in Sri Lanka

--Manuscript Draft--

**Manuscript Number:**	PGPH-D-26-00295R1
**Article Type:**	Research Article
**Full Title:**	Challenges in strengthening multi-sectoral action for optimum preparedness and response for public health emergencies in Sri Lanka
**Short Title:**	Challenges in strengthening multisectoral action in public health emergencies in Sri Lanka
**Corresponding Author:**	Suriyakumar Mahendra Arnold, MBBS, MSc, MBA, AAL, MD<br>Ministry of Health: Government of Sri Lanka Ministry of Health Sri Lanka<br>Colombo 10, SRI LANKA
**Order of Authors:**	Suriyakumar Mahendra Arnold, MBBS, MSc, MBA, AAL, MD
	Pasyodun Koralage Buddhika Mahesh, MBBS, MSc, MD
	Dakshila Indikadahena Galappatti, MBBS, MSc, MD
	Asela Gunawardena, MBBS, MSc
**Keywords:**	public health emergencies; multisectoral action; challenges for collaboration; NPHA; policy challenges; policy deficiencies; Sri Lanka
**Abstract:**	Effective coordination between health and other sectors facilitate a better preparedness and response for public health emergencies (PHEs). Sri Lanka is a lower-middle income island nation that often contends with multiple public health emergencies such as floods and disease epidemics. In preparing and responding to these PHEs, different units of the Ministry of Health (MOH) address the functions of a typical National Public Health Agency in Sri Lanka. This study was done to describe current approaches to multisectoral action and to explore the challenges to multisectoral action involving the MOH in preparedness for and response to PHEs in Sri Lanka, with particular emphasis on policies, agreements and legislation. The study included a risk assessment, stakeholder-mapping, a desk review of literature and documents available with institutions which are potential stakeholders as well as qualitative analysis of key informant interviews (KIIs) covering the nine provinces of Sri Lanka. The risk assessment was done to identify potential PHEs while the stakeholder grid was prepared to identify key stakeholders and determine their level of engagement. The desk review aimed to identify the deficiencies of the existing major policy documents for multisectoral action. The study team conducted 51 KIIs using a semi-structured questionnaire with purposively selected respondents from March to June 2025 The US CDC model on public health linkages served as the conceptual framework, including four domains: 1) policies/agreements/legislation, 2) infrastructure, 3) governance/funding/logistics, and 4) people-organization culture. Thematic coding analysis was performed to synthesise challenges under these domains. The main challenges imposed by policy deficiencies to multisectoral action involving the MOH included: lack of inclusive engagement of stakeholders in policy formulation leading to fragmented implementation, not having a strong policy architecture for collaboration, non-adaptive policies and vague terminology giving rise to uncertainties of interpretations. Challenges imposed by policy-deficiencies on other three domains included: sub-optimum data management, delayed fund mobilization, lack of mediation of activities of NGOs, less-customized risk communication, and ad-hoc collaborative efforts between stakeholders. To address these challenges, the study recommends adapting existing policy documents, clarifying decentralization and sub-national implementation arrangements, establishing inter-stakeholder operational guidelines, and strengthening intersectoral awareness of roles and communication mechanisms.
**Opposed Reviewers:**	
**Additional Information:**	

Powered by Editorial Manager® and ProduXion Manager® from Aries Systems Corporation

Question	Response
**Financial Disclosure**<br><br>Enter a financial disclosure statement that describes the sources of funding for the work included in this submission and the role the funder(s) played. This includes grants and any commercial funding of the work or authors.<br><br>This statement will be typeset if the manuscript is accepted for publication.<br><br>*Please review the submission guidelines and the instructions link below for detailed requirements and guidance.*	This work received financial support from the Alliance for Health Policy and Systems Research. [203591828, S.M]. The Alliance is able to conduct its work thanks to the commitment and support from a variety of funders. These include our long-term support from the Swedish International Development Cooperation Agency (Sida) and the Norwegian Agency for Development Cooperation (Norad), as well as designated funding for specific projects within our current priorities. This research was supported through funding provided to WHO Health Emergencies, with thanks to the Government of Germany and the Federal Ministry of Health for their contribution. For the full list of Alliance donors, please visit https://ahpsr.who.int/about-us/funders. The funders had no role in study design, data collection and analysis, decision to publish, or preparation of the manuscript.
**Competing Interests**<br><br>On behalf of all authors, disclose any competing interests that could be perceived to bias this work.<br><br>This statement will be typeset if the manuscript is accepted for publication.<br><br>*Please review the instructions link below and PLOS Global Public Health's competing interests policy to determine what information must be disclosed at submission.*	The authors have declared that no competing interests exist.
**Data Availability** <br>Before publication, Authors are required to make fully available and without restriction all data underlying their findings. Sharing the data underlying your findings increases the visibility, credibility, and impact of your work, while supporting transparency and future scientific discovery. Please see our PLOS Data Policy page for detailed information on this policy. <br>A **Data Availability Statement**, detailing where the data can be accessed, is required at first	All data used for this manuscript is available in the manuscript. However, identity of the respondent will not be revealed for key informant interviews and personal opinions made.

submission. Insert your Data Availability Statement in the box below. <br>Please see the data reporting section of our submission guidelines for instructions on what you need to include in your Data Availability Statement. Two examples of acceptable Data Availability Statements are included here for reference: The minimal data set [and accompanying code, where generated] is available at [repository name] via [DOI hyperlink]. No data was generated by this study. The following existing data sources were used: [dataset name] from [dataset location] available via [web address or DOI hyperlink] <i>PLOS allows rare exemptions to address legal and ethical concerns. If you have legal or ethical restrictions, please use the box below to detail these in full sentences for the Journal team to consider.</i></p> This statement will be typeset if the manuscript is accepted for publication.	

22nd January 2026

Editor
PLOS Global Public Health

Dear Editor,

Submission of manuscript

We are pleased to submit our manuscript entitled ***"****Challenges in strengthening multi-sectoral action for optimum preparedness and response for public health emergencies in Sri Lanka****"*** for consideration in PLOS Global Public Health**.**

This work is original, has not been published elsewhere, and is not under consideration by another journal. All authors have approved the manuscript and its submission. Ethical approval was obtained from Ethics Review Committee, National Institute of Health Sciences, Kalutara, Sri Lanka.

This work received financial support from the Alliance for Health Policy and Systems Research. [203591828, S.M]. The Alliance is able to conduct its work thanks to the commitment and support from a variety of funders. These include our long-term support from the Swedish International Development Cooperation Agency (Sida) and the Norwegian Agency for Development Cooperation (Norad), as well as designated funding for specific projects within our current priorities. This research was supported through funding provided to WHO Health Emergencies, with thanks to the Government of Germany and the Federal Ministry of Health for their contribution. For the full list of Alliance donors, please visit https://ahpsr.who.int/about-us/funders. The funders had no role in study design, data collection and analysis, decision to publish, or preparation of the manuscript.

We believe this study will be of interest to the journal's broad global public health readership.
Thank you for your consideration.

Sincerely,

Dr S.M. Arnold
Ministry of Health, Sri Lanka
E mail: mahendra_arnold@yahoo.com

Manuscript
Click here to access/download;Manuscript;Manuscript Final - 2026.01.23.docx

Title:

Challenges in strengthening multi-sectoral action for optimum preparedness and response for public health emergencies in Sri Lanka

Authors:

Suriyakumar Mahendra Arnold

Ministry of Health, Sri Lanka

Pasyodun Koralage Buddhika Mahesh

Ministry of Health, Sri Lanka

Dakshila Indikadahena Galappatti

Ministry of Health, Sri Lanka

Asela Gunawardena

Ministry of Health, Sri Lanka

Challenges in strengthening multi-sectoral action for optimum preparedness and response for public health emergencies in Sri Lanka

Abstract

Effective coordination between health and other sectors facilitate a better preparedness and response for public health emergencies (PHEs). Sri Lanka is a lower-middle income island nation that often contends with multiple public health emergencies such as floods and disease epidemics. In preparing and responding to these PHEs, different units of the Ministry of Health (MOH) address the functions of a typical National Public Health Agency in Sri Lanka. This study was done to describe current approaches to multisectoral action and to explore the challenges to multisectoral action involving the MOH in preparedness for and response to PHEs in Sri Lanka, with particular emphasis on policies, agreements and legislation. The study included a risk assessment, stakeholder-mapping, a desk review of literature and documents available with institutions which are potential stakeholders as well as qualitative analysis of key informant interviews (KIIs) covering the nine provinces of Sri Lanka. The risk assessment was done to identify potential PHEs while the stakeholder grid was prepared to identify key stakeholders and determine their level of engagement. The desk review aimed to identify the deficiencies of the existing major policy documents for multisectoral action. The study team conducted 51 KIIs using a semi-structured questionnaire with purposively selected respondents from March to June 2025 The US CDC model on public health linkages served as the conceptual framework, including four domains: 1) policies/agreements/legislation, 2) infrastructure, 3) governance/funding/logistics, and 4) people-organization culture. Thematic coding analysis was performed to synthesise

challenges under these domains. The main challenges imposed by policy deficiencies to multisectoral action involving the MOH included: lack of inclusive engagement of stakeholders in policy formulation leading to fragmented implementation, not having a strong policy architecture for collaboration, non-adaptive policies and vague terminology giving rise to uncertainties of interpretations. Challenges imposed by policy-deficiencies on other three domains included: sub-optimum data management, delayed fund mobilization, lack of mediation of activities of NGOs, less-customized risk communication, and ad-hoc collaborative efforts between stakeholders. To address these challenges, the study recommends adapting existing policy documents, clarifying decentralization and sub-national implementation arrangements, establishing inter-stakeholder operational guidelines, and strengthening intersectoral awareness of roles and communication mechanisms.

Keywords: Public Health Emergencies; multisectoral action; challenges for collaboration; NPHA; policy challenges; policy deficiencies; Sri Lanka

Introduction

Public Health Emergencies (PHEs) encompass acute events – natural or manmade – that challenge health systems due to their scale, timing and unpredictability(1,2).The COVID-19 pandemic shed light on the importance of rapid and coordinate health system responses to PHEs. PHEs threaten lives and disrupt economies. It is therefore essential to effectively organize health systems to

enhance their response capacity and resilience. PHEs pose direct and indirect threats to all building blocks of the health system (2,3). Multisectoral action is necessary to strengthen all building blocks. This study examines approaches to and challenges in multisectoral action for enhancing health system preparedness for PHEs, drawing on experiences in Sri Lanka.

Sri Lanka, a lower-middle income country with a population of approximately 22 million is no exception to the impact of PHEs with periodic events like floods, adverse weather conditions, and epidemics including dengue and COVID-19. The World Health Organization (WHO) states that multisectoral actions can strengthen "country ownership, accountability, stewardship of resources, and organizational effectiveness around health emergency preparedness, readiness and response"(4). Further, when multisectoral action is taken during the preparatory phase rather than the response phase, the benefits are greater (4).

Many models or frameworks have been developed to depict the level of preparedness and response for PHEs through multisectoral action (3,5–10). The multisectoral preparedness coordination framework highlights key elements of such an approach (4). US-CDC has proposed a comprehensive framework to highlight "information on practical approaches public health leaders may consider using to strengthen linkages and their enablers for improved public health impact in their own national contexts" (11). These linkages have been defined as "practical, replicable activities or actions that facilitate collaboration between public health functions or organizations to improve public health impact"(11) In this model, the linkages have been elaboratively categorized under five levels; functional, multifunctional, multisectoral, multilevel and international (11). Four enablers have been mentioned in this model: "policies, agreements and

legislation”, “infrastructure”, “governance, funding and logistics” and “people and organizational culture”(11)

While all these four enabler-domains are important, “policies, agreements and legislation” drive the coordination of multisectoral response in PHEs. Out-of-date policies and lack of understanding on the available policies have been identified as challenges to optimum response in PHEs in the global literature(2). Meanwhile, characteristics of a successful health system include resilience and the capacity to learn from experience, feeding knowledge and data back into the policy (12).

Literature on “policies, agreements and legislation” for multisectoral action for PHEs in Sri Lanka is scarce and has not been consolidated. Thus, the current study aims to describe the current risk of PHEs in Sri Lanka, and to examine the approaches and challenges of multisectoral action in response efforts, with particular emphasis on policies, agreements, and legislation.

Methods

The study included a national risk assessment for PHEs, stakeholder mapping, desk review, and a qualitative study covering respondents in all 25 districts and nine provinces of Sri Lanka (13,14).

The risk assessment was done to determine the priority PHE risks presently faced by Sri Lanka, which warrant multisectoral action. This was done with secondary data available at Disaster Preparedness and Response Division, Provincial Directorate of Health Services and Regional

Directorate of Health Services for the last 10 years as well as with inputs from the experts in those institutions. The secondary data included preparedness plans for PHEs and reports available on response phases.

A stakeholder mapping exercise was conducted to identify the major stakeholders of multisectoral action and assess their degree of engagement in PHEs. The stakeholders who have been involved or who are expected to be involved in potential events were also documented, through a stakeholder mapping activity, following guidance from the WHO "Multisectoral preparedness coordination framework" (4). Each stakeholder was rated as "high or low" for the two domains of "impact" and "influence" (15); "impact" was defined as "how much of an impact does the stakeholder have on preparedness and response on PHEs" and "influence" was defined as "how much authority does the stakeholder have on preparedness and response on PHEs" (15).Those with high levels of both were identified. Level of engagement was categorized as: consult (high interest-high influence), convince (high influence- low interest), update (high interest- low influence) and monitor (low influence- low interest) (15).Co-authors discussed these assignments for consensus, and where needed, also consulted the relevant officers. Participants for KIIs were recruited from the institutions identified as having "consult" and "convince" levels of engagement (15).

A desk review was conducted to collate evidence for facilitators and barriers for multisectoral action and its governance. This included an online search in PubMed and Google Scholar and a review of reports on preparedness planning and responses from the various stakeholders. Expert feedback provided recommendations for additional grey literature and policy documents to include for review. In PubMed, subject heading and keyword searches were done. The subject heading

search was done by exploring the relevant MeSH terms and keywords. The complete search strategy is shown in Annex 1. The reports that were reviewed included documents related to PHE preparedness and response activities available with stakeholders.

Interviews were conducted with stakeholders with experience working in the institutions that were determined to have both high impact and influence in multisectoral action (16–20). Altogether, the team conducted 51 KIIs between 1st April and 31st May 2025 following recommended methodology (16–20). Participants included different team members involved in: policy planning, implementation, research, administration, training, and financing. Furthermore, the participants covered both government sector and non-governmental sectors, such as the Sri Lanka Red Cross. In the government sector, stakeholders from national, provincial, district and divisional levels were included. Both curative and preventive sectors as well as health and non-health sectors were covered. The institutional breakdown of the 51 KIIs conducted is shown below in Table 1.

Table 1: KIIs conducted in different institutions

**Type of institution**	**Number of KIIs**
Line Ministry Health Institutions	05
Offices of Provincial and Regional Directors of Health Services (PDHS & RDHS)	28
Offices of Medical Officer of Health (MOH)	04
Curative Health Institutions	02
Disaster Management Centre	03

Provincial Council, District Secretariat, Divisional Secretariat	04
Security forces	02
Academia	01
NGOs	02
Total	51

Representation of the Provincial/ District health staff in the KIIs is shown in Annex 2.

Purposive sampling was done by discussing with staff members of selected institutions. A semi-structured questionnaire was prepared. The contents of the questionnaire were based on the four-domains of the US CDC framework. In this model, four enablers of public health linkages have been proposed as: 1). Policies, agreements and legislation, 2). Infrastructure, 3). Governance, funding and resources and 4). People and organizational culture. Interviews were done by study investigators with one being the moderator and another being a note taker. Informed written consent was obtained from participants including for audio recording of interviews. The audio recordings were transcribed following principles from Baily et al (2008) and spot-checked for quality. Data were analysed and interpreted concurrently using a convergent-parallel design to integrate findings across methods(21). Thematic analysis was done under the domains of the US CDC framework (22).

Ethics approval was obtained from the Ethics Review Committee of National Institute of Health Sciences (NIHS/ERC/24/77R).

Results

The results are presented in the order of: 1. risk assessment and stakeholder mapping, 2. desk review findings including the current structure of delivery of NPHA functions in Sri Lanka, and 3. findings from the KIIs.

Actual and potential PHEs of Sri Lanka and stakeholder-grid

The risk assessment revealed that PHEs in the previous 10 years included: COVID-19, dengue outbreaks, floods, landslides, explosions, and events due to adverse weather conditions. In addition, potential future event risks include: tsunami, other disease epidemics, chemical accidents, and recurrences of those that have been reported within last 10 years. The stakeholder-grid that was prepared to identify the organizations from which to draw the KII respondents is shown below in Table 2.

Table 2: Stakeholder grid

Organization	Type of influence	Level of impact	Level of influence	Level of engagement
Disaster Management Centre	-Policy formulation<br>-Administration<br>-Coordination	High	High	Consult

Disaster Preparedness and Response Unit- Line Ministry	-Policy formulation<br>-Coordination	High	High	Consult
Offices of Provincial Director of Health Services	-Implementation<br>-Technical	High	High	Consult
Offices of Regional Director of Health Services	-Implementation<br>-Technical	High	High	Consult
Epidemiology Unit	-Policy formulation<br>-Technical	High	High	Consult
Family Health Bureau	-Policy formulation<br>-Technical	High	High	Consult
Health Promotion Bureau	-Policy formulation<br>-Technical	High	High	Consult
Environmental & Occupational Health Unit	-Policy formulation<br>-Technical	High	High	Consult
National Dengue Control Unit	-Policy formulation<br>-Technical	High	High	Consult
Anti Malaria Campaign	-Policy formulation	High	High	Consult

	-Technical			
Provincial Councils	-Administration<br>-Implementation<br>-Financial	High	High	Consult
District Secretariate	-Administration<br>-Implementation<br>-Financial	High	High	Consult
Divisional Secretariate	-Administration<br>-Implementation	High	High	Consult
Offices of Medical Officers of Health	-Implementation<br>-Technical	High	High	Consult
Curative institutions	-Implementation<br>-Supportive	High	High	Consult
Security forces	-Supportive<br>-Implementation	High	High	Consult
Non-Governmental Organizations	-Supportive<br>-Financial	High	Low	Convince
Universities	-Technical	High	Low	Convince

Except for the NGOs and universities, all other institutions mentioned in the above table were categorized as having a high level of impact and influence and thus being in the “consult” level of engagement. The former two were assigned the “convince” level of engagement(15).

From the desk review and the discussions with experts in the institutions, eight policy documents related to multisectoral action were identified. These eight policy documents were categorized as having: 1) general components of disasters, 2) health-related disasters as the main focus, 3) risk communication, and 4) security as the main focus. An analysis of the policy documents related to multisectoral actions in PHEs and their gaps are shown in Table 4 with a summary of each provided below.

The Disaster Management Act gave the opportunity to form the Disaster Management Centre (DMC) which is the lead agency for coordinating preparedness, mitigation, and recovery efforts in emergencies/disasters(23). The National Disaster Management Plan (NDMP) 2023-2030 serves as Sri Lanka's comprehensive framework for disaster risk management(24). It encompasses various hazards, including health emergencies such as epidemics and pandemics. The National Emergency Operation Plan (NEOP), provides a comprehensive framework for coordinating emergency responses across various hazards aiming to enhance national resilience through multi-sectoral coordination, early warning systems, and standardized response protocols(25). The Sri Lanka National Health Policy (2016-2025) is built upon the concept of "Universal Health Coverage" (UHC), aiming to ensure accessibility to quality services with financial protection. The National Health Strategic Master Plan 2016–2025 underscores a commitment to addressing emerging health challenges, improving infrastructure, and ensuring compliance with international health standards, including the IHR 2005(26). The National Risk Communication Strategic Plan of Sri Lanka aims to strengthen Sri Lanka's capacity to provide accurate information, counter misinformation, and build public trust in health interventions. The Public Security Ordinance No.

25 of 1947 is a key legal instrument in Sri Lanka that enables the integration of public health and security authorities during emergencies, including pandemics and disease outbreaks(27). Although the primary mandate of Sri Lanka Police Ordinance No. 16 of 1865 is on law enforcement and community policing, its provisions offer potential leverage points for extending the role of the police force in PHEs(28).

Table 4: Existing policy documents and their gaps

**Policy document**	**Deficiencies**
**Policies related to general components of disasters as the main focus**	
1.Disaster Management Act No. 13 of 2005	Designed mainly for natural disasters and not for disease epidemics and pandemics.<br>Coordination on preparedness and response of DMC based on disease surveillance is not optimal with health and animal sector.
2. National Disaster Management Plan, 2023-2030	No policy mandate for real time integrated Disease Surveillance System available, which hinders early risk mitigation and prevent health emergencies.
3. National Emergency Operation Plan (NEOP)	IHR requires detailed capacities in surveillance and laboratory diagnostics. The NEOP lacks explicit references to national laboratory networks or protocols for epidemiological surveillance.
**Policies related to disasters with health as the main focus**	
4. National Health Policy 2016 – 2025	Institutionalization of multisector coordination is weak in the policy text. While the policy endorses intersectoral action, it stops

	short of prescribing statutory or permanent governance arrangements such as having district multisector fora with defined mandates and reporting.<br><br>Subnational operational capacity and decentralisation not fully detailed - the Policy's national orientation needs clearer provisions for district/divisional roles and resourcing.
5. National Health Strategic Master Plan 2016 – 2025	Human Resources for Health (HRH) strategies exist but implementation gaps like uneven distribution, retention problems in rural/estate areas, unclear task-shifting policies persist. Strengthening HRH was repeatedly identified as a constraint.<br><br>The plan references eHealth but interoperability and nationwide data governance were not fully implemented. This created fragmented records and poor information flows.<br><br>The Master Plan recognises the private sector but lacks a strong, operational regulatory framework and clear engagement models to align private provision with public goals.
**Policies related to risk communication**	

6. National Risk Communication Strategic Plan for Public Health Emergencies in Sri Lanka	Although the plan emphasizes community engagement, there is little indication of tailored messaging for different socio-cultural groups like urban slum dwellers and estate communities beyond general language translation.
**Policies related to security**	
7.Public Security Ordinance	There is no dedicated mechanism to ensure routine collaboration between the Ministry of Health and the Ministry of Defense for health emergency readiness.
8.Sri Lanka Police Ordinance	The Police Ordinance does not explicitly include public health as an area of responsibility, leading to potential legal uncertainties when police are called upon for health-related duties.

Current structure of delivery of NPHA functions in Sri Lanka

Following the desk review and discussions with the institutions, the current mechanisms of delivering NPHA functions were summarized. Currently, there is no single entity named as a National Public Health Agency (NPHA) in Sri Lanka. The central Ministry of Health issues guidelines while the provincial, district, and divisional level stakeholders contribute to the implementation. At the central level, the Quarantine Unit and Epidemiology Unit function as co-

national focal points under the International Health Regulations IHR 2005 while the latter leads in disease surveillance as well. Health sector preparedness and response in general are coordinated by the Disaster Preparedness and Response Division of Ministry of Health. There are other specific control programs like the National Dengue Control Unit and the National Tuberculosis and Chest Diseases Control Programme. The non-health stakeholders like the Provincial Councils, Local Government bodies, District Secretariates and Divisional Secretariates play critical coordination and response-implementation roles in the preparedness and response in PHEs.

Building on the limitations identified in the desk review, challenges to multisectoral action as suggested by the key informants are shown below in Table 3. The experts pointed out eight challenges in relation to policy-agreement-legislation domain, highlighting deficiencies in policy focus, priorities, architecture, implementation, and monitoring. The second domain that covered physical and digital infrastructure yielded seven challenges that covered aspects like lack of common physical infrastructure as well as sub-optimal data management. Challenges in relation to fund mobilization, donation processes, and NGO coordination were highlighted for the domain on "governance-funding and resources". Six challenges that covered aspects like training fora, scopes of work, standard operating protocols, sustainability, and integration of social media were described under the domain on "people-organization culture".

Table 3: Challenges as suggested by the Key Informants

**Policies- agreements-legislation domain**	**Infrastructure domain**

• Major policy and legislative instruments emphasize response actions more than preparedness<br>• Priorities are not aligned across sectors in relation to PHEs<br>• No strong policy architecture is present for collaboration between actors<br>• NGOs have to fulfil their own funders' requirements<br>• It is often necessary to go through default hierarchies of the administration in incident commanding system, limiting efficiency<br>• There is a lack of documented policies on data sharing<br>• There is a lack of monitoring mechanisms for multisectoral action for PHEs<br>• There is a lack of policies on documentation in relation to preparedness and response for PHEs	• Limited identification of a common infrastructure for the response phase was noted<br>• Non-availability of an integrated laboratory network was found<br>• There is a lack of resource mapping across different sectors<br>• Fragmented data gathering is observed leading to duplication of efforts<br>• There is no integrated database<br>• Discrepancies between data collected from different sources are noted<br>• Minimum use of digital technology in data management is seen, leading to more manual efforts
**Governance, funding and resources domain**	**People and organization culture domain**
• Non-availability of a separate fund to mobilize in PHEs was noted	• No common training for a are found

• Undue delays in fund mobilization are experienced<br>• There is no mechanism to jointly monitor the functions and processes related to PHEs<br>• Delays in mobilizing and redistribution of resources in the response phase are noted<br>• Duplication is found in activities done by NGOs in some instances<br>• There is lack of clarity on donation processes	• Lack of awareness on others' scope in PHEs was noted<br>• Lack of clarity on SOPs on other stakeholders was noted<br>• Coordination often depends on personal connections that may not sustain with changes in human resources<br>• Non-health focal points sometimes lack awareness on and knowledge of health system operations and needs<br>• Non-availability of a system to use the beneficial effects and to reduce harmful influence of social-media was found

By collating the evidence from the literature review, desk review and KIIs, the main challenges in relation to policy deficiencies were synthesized. A key challenge identified was the lack of inclusive engagement of stakeholders in policy formulation, which has led to fragmented implementation. This challenge is further exacerbated by the absence of a strong policy architecture to support collaboration between actors and to enable timely adaptation to meet changing needs. As a result, outdated and uncoordinated policies create uncertainty about how provisions apply, particularly within a decentralized system, and contribute to inefficiencies in administrative, operational, and financial processes during PHEs. Another challenge identified is

the absence of policy provisions for a common database, limiting the opportunities for data integration across sectors. The lack of an established laboratory network represents a manifestation of this gap. Additionally, vague terminology within security polices was highlighted as another challenge as it gives rise to uncertainties in interpretations.

Discussion

This is the first documented qualitative study done in Sri Lanka at the national level on approaches and challenges for effective multisectoral action in PHEs. The findings revealed current PHE risks, identified key stakeholders for multisectoral action in PHEs in Sri Lanka, and explored potential challenges in relation to the four domains of the US CDC model: policies/ agreements/legislations, infrastructure, governance/funding/logistics, and people/ organizational culture. Our stakeholder mapping exercise identified a large number (n=16) of categories of stakeholder institutions that were considered "high" in terms of their influence and impact, and in totality, represent a complex environment of stakeholders that require coordination and consultation. The findings suggest that there is no uniform framework that units are using for multisectoral governance.

Under "policies, agreements, and legislation", the major challenges identified included policy deficiencies related to policy architecture, policy inclusiveness, policy adaptation, and vague policy terminology. These deficiencies are interconnected and contribute to the root cause of many other challenges in achieving multisectoral action for health. For example, non-adaptive and out-of-date policies hinder optimal functioning and efficient processes within decentralized governance structures. In addition, vague terminologies in legal policy documents, particularly around security-related policies, create uncertainties in interpretation and constrains their application in supporting PHE response efforts. Emphasizing the importance of clarifying legal

provisions, the National Summit on Public Health Legal Preparedness in 2007 highlighted the need for ensuring legal preparedness in the context of PHEs (24). Many of the other policy-related challenges identified in this study are aligned with those have been reported globally as well as in regional literature, including in a comparative study done across nine countries (29–33).The reported literature shows the deficiencies of the health policy processes hindering aspects like inclusiveness and meaningful engagement in PHEs (29–33).

The identified policy deficiencies lead to difficulties in relation to other domains of the US CDC framework as well. One example is fragmented coordination of the utilization of digital infrastructure (i.e. the infrastructure domain) leading to duplications in data management. For example, in a flood event, data are collected by the different units of the Ministry of Health itself using different data templates that creates unnecessary workloads for ground-level staff. In epidemic events, identified deficiencies give rise to undue delays in funding mobilization that include transferring of funds along traditional hierarchical levels rather than having a more agile system in place as part of preparedness activities. In general, the absence of policy provisions to mandate and enable multisector collaboration and coordination, shared preparedness training platforms, and a common understanding of roles, scope of work, and resources across sectors, contributes to inefficiencies in response efforts. For example, health units may be unable to leverage support from non-health stakeholders during the response phase if roles and responsibilities are not clearly understood. Hence addressing the policy domain as a primary driver for multisectoral action, serves to intervene in challenges under other domains as well.

The importance of policy provisions is further highlighted by the absence of a provision to have a structural coordinating agency like an NPHA. Coordinating agencies are particularly critical as poor stakeholder engagement leads to fragmentation of services in relation to preparedness and response to PHEs(32). Even though the services related to PHEs are delivered from different units within the Ministry of Health, this fragmentism leads to duplication anad gaps in some control measures. Similarly, the lack of coherent implementation and central monitoring lead to misalignment of priorities across organizations.

Under infrastructure, the major challenges identified in the study were non-identification of common physical infrastructure and sub-optimal data management. Exacerbated by the absence of an integrated real-time database and lack of documented policies on data sharing, this creates a vicious cycle leading to poor data integration and management. This challenge was also identified in a mixed methods study in Ethiopia where limited digital infrastructure and incompatibility of data formats were identified as barriers to effective data coordination (33). Sri Lanka should develop integrated unified databases to be used in PHEs to integrate operational data. Currently such databases – linking curative and preventive efforts – are found in relation to communicable conditions like dengue and tuberculosis. Once developed, such a platform can integrate health surveillance data with data from other sectors facilitating initiatives like the One Health approach (34). The absence of a laboratory network, which was highlighted as another challenge, can also be addressed by a unified database and real-time data exchange. Thus, the main national laboratory should be linked with those at peripheral levels and this network could be incorporated to the integrated database with surveillance data.

For the “governance-funding and resources” domain, several financial difficulties were highlighted. Given this context, lack of an anticipatory pre-arranged financing mechanism makes the country more vulnerable(35). Outdated financial procedures should be reviewed and policy adaptations undertaken. Under the domain of people and organization culture, it was elicited was that most connections between organizations are on a personal level and not due to system-driven mechanisms, policies, or practices. In contrast, the better approach is to have well-laid system-level planning which is more sustainable(36). A multi-site qualitative study has shown that, although ad-hoc interactions between professions may provide some benefit, it would not give rise to “systematic forms of collaboration”(37).

The WHO has emphasized the value of proactive considerations and far-sighted policies over limited response measures (4). Within this context, the way forward must include several considerations. Policy adaptations with timely revision of currently existing policy documents are one such worthy endeavour. Within this, defining clearer terms in relation to decentralization and sub-national implementation are recommended. Having specific inter-stakeholder operational guidelines, raising intersectoral awareness on mutual scopes, and better sectoral dialogues are other key considerations. Once the policy domain is addressed, it will facilitate addressing the challenges of the other three domains as well. Challenges like the presence of data discrepancies, fragmented data gathering, absence of a common database can be solved once the data sharing provisions are thus streamlined through policies. At the same time, the funding related challenges mentioned under domain three can also be addressed and the human resource related challenges such as training and raising the awareness will also be facilitated once the policies are clearer with the policy reforms.

There were several limitations of the study. The challenges identified in the study were based on the subjective experiences and opinions of individuals and may be informed by one event or limited to one setting. To mitigate the potential for bias, a larger number of KIIs across a range of organizations – identified in the stakeholder mapping exercise – were conducted to ensure the inclusion of diverse perspectives and to strengthen the robustness of emergent themes. Furthermore a consistent prioritization technique was used that helped standardize inputs more representative.

Conclusions and recommendations

This novel study identified critical challenges with multisectoral collaboration in Sri Lanka, involving policy, infrastructure, funding, and people organization culture. We found that services related to PHEs are delivered by different units in Sri Lanka without the existence of an NPHA entity to support coordination. Policy deficiencies give rise to challenges including lack of inclusive engagement of stakeholders in policy formulation leading to fragmented implementation, absence of a strong policy architecture for collaboration, lack of policy adaptation and vague terminology giving rise to uncertainties in interpretations. In addition, policy deficiencies impose challenges for the other three domains of the US CDC framework as well. These include: sub-optimal data management, delayed fund mobilization, suboptimal coordination of NGOs, and ad-hoc collaborative efforts between stakeholders. To address these challenges, policy adaptation of existing policy documents, defining clearer terms in relation to decentralization and sub-national implementation (i.e. clarifying roles, authority, and responsibilities), having specific inter-stakeholder operational guidelines, raising intersectoral awareness on mutual scopes and system-

driven intersectoral communication are recommended as a first step toward a truly multisectoral approach to preapredness and response to PHEs in Sri Lanka.

Acknowledgements

This work received technical support from the Alliance for Health Policy and Systems Research and the WHO Health Emergencies Programme. The authors extend their sincere gratitude to Dr Kumanan Rasanathan, Dr Ana Lorena Guerrero Torres, Dr Ruwan Ratnayake and Dr Alexandra Zuber for their technical guidance throughout the research project.

References

1. Nelson C, Lurie N, Wasserman J, Zakowski S. Conceptualizing and defining public health emergency preparedness. Am J Public Health. 2007 Apr;97 Suppl 1(Suppl 1):S9-11.
2. Khatri RB, Endalamaw A, Erku D, Wolka E, Nigatu F, Zewdie A, et al. Preparedness, impacts, and responses of public health emergencies towards health security: qualitative synthesis of evidence. Archives of Public Health. 2023 Nov 30;81(1):208.
3. Olu O. Resilient Health System As Conceptual Framework for Strengthening Public Health Disaster Risk Management: An African Viewpoint. Front Public Health. 2017 Sep 28;5.
4. World Health Organization. Multisectoral Preparedness Coordination Framework: best practices, case studies and key elements of advancing multisectoral

coordination for health emergency preparedness and health security. Geneva; 2020.

5. Ario AR, Makumbi I, Bulage L, Kyazze S, Kayiwa J, Wetaka MM, et al. The logic model for Uganda’s health sector preparedness for public health threats and emergencies. Glob Health Action. 2019;12(1).

6. Hong Y, Zhao J, Zhang Y, Su Q. Motivating public health emergency preparedness cooperative behaviors: based on the expectancy disconfirmation model. J Health Organ Manag. 2023;37(6–7).

7. Lowe AE, Kraft C, Kortepeter MG, Hansen KF, Sanger K, Johnson A, et al. Developing a Rapid Response Single IRB Model for Conducting Research during a Public Health Emergency. Vol. 20, Health Security. 2022.

8. Stoto MA, Nelson C, Savoia E, Ljungqvist I, Ciotti M. A Public Health Preparedness Logic Model: Assessing Preparedness for Cross-border Threats in the European Region. Health Secur. 2017;15(5).

9. Eerens D, Hrzic R, Clemens T. The architecture of the European Union’s pandemic preparedness and response policy framework. Eur J Public Health. 2023;33(1).

10. Yaylali E, Ivy JS, Taheri J. Systems engineering methods for enhancing the value stream in public health preparedness: The role of Markov models, simulation, and optimization. Public Health Reports. 2014;129.

11. United States Centers for Disease Control and Prevention. Improving Linkages between Public Health Functions and Organizations- A framework for National Public Health Institutes. 2024.

12. Mills A. Health Care Systems in Low- and Middle-Income Countries. New England Journal of Medicine. 2014 Feb 6;370(6):552–7.

13. Abusayeed MH, Rampatige RGV, Peiris SL, Galappathi H. A Qualitative Assessment of the Health Information System in Sri Lanka using HMN assessment tool. Sri Lanka Journal of Bio-Medical Informatics. 2010;1(2).

14. Prakash SL, Abeykoon P, Niwarthana G, Malshan B, Nanayakkara S, Nanayakkara J, et al. Community participating ‘smoke free zones’ against second hand smoking in Sri Lanka; present and future. Tob Induc Dis. 2021;19(1).

15. World Health Organization. Stakeholder mapping and engagement- WHO SEARO Salt Reduction Toolkit.

16. Zacharia B, Pai PK, Paul M. Focus Group Discussion as a Tool to Assess Patient-Based Outcomes, Practical Tips for Conducting Focus Group Discussion for Medical Students—Learning With an Example. J Patient Exp. 2021;8.

17. Khan NA, Abedin S. Focus Group Discussion. In: Principles of Social Research Methodology. 2022.

18. Ahmed SK. The pillars of trustworthiness in qualitative research. Journal of Medicine, Surgery, and Public Health. 2024;2.

19. Adler RH. Trustworthiness in Qualitative Research. Journal of Human Lactation. 2022;38(4).

20. Kakar ZUH, Rasheed R, Rashid A, Akhter S. CRITERIA FOR ASSESSING AND ENSURING THE TRUSTWORTHINESS IN QUALITATIVE RESEARCH. International Journal of Business Reflections. 2023;4(2).

21. Ozawa S, Pongpirul K. 10 best resources on … mixed methods research in health systems. Health Policy Plan. 2014 May 1;29(3):323–7.

22. Bingham AJ. From Data Management to Actionable Findings: A Five-Phase Process of Qualitative Data Analysis. Int J Qual Methods. 2023 Jan 10;22.

23. Disaster Managemnet Centre. Disaster Management Act No.13 of 2005 [Internet]. 2005 [cited 2026 Jan 16]. Available from: https://www.dmc.gov.lk/images/DM_Act_English.pdf

24. Disaster Management Centre. The National Disaster Management Plan (NDMP) 2023-2030 [Internet]. 2023 [cited 2026 Jan 16]. Available from: https://www.dmc.gov.lk/images/pdfs/NDMP_V2_withCoverV2.pdf

25. Disaster Management Centre. National Emergency Operation Plan [Internet]. 2017 [cited 2026 Jan 16]. Available from: https://www.dmc.gov.lk/images/pdfs/NEOP/NEOP_English.pdf

26. Ministry of Health Sri Lanka. National Health Strategic Master Plan [Internet]. 2016 [cited 2026 Jan 16]. Available from: https://www.health.gov.lk/moh-pages/health-master-plan-2016-2025/

27. Government of Sri Lanka. Public Security Ordinance [Internet]. 1947 [cited 2026 Jan 16]. Available from: https://www.lawnet.gov.lk/wp-content/uploads/leg_enact_1981/1981Y3V51C.html

28. Government of Sri Lanka. Police Ordinance [Internet]. 1865 [cited 2026 Jan 16]. Available from:

https://www.vertic.org/media/National%20Legislation/Sri%20Lanka/LK_Police_Ordinance.pdf
29. Shah I, Elahi N, Alam A, Dawar S, Dogar AA. Institutional arrangement for disaster risk management: Evidence from Pakistan. International Journal of Disaster Risk Reduction. 2020 Dec;51:101784.
30. El-Jardali F, Fadlallah R, Daher N. Multi-sectoral collaborations in selected countries of the Eastern Mediterranean region: assessment, enablers and missed opportunities from the COVID-19 pandemic response. Health Res Policy Syst. 2024 Jan 24;22(1):14.
31. Miqdadi R, Hamdan M. Key stakeholders' perspectives of public health emergency preparedness in Palestine: A qualitative exploratory study. Public Health. 2024 Dec;237:232–7.
32. Masefield SC, Msosa A, Chinguwo FK, Grugel J. Stakeholder engagement in the health policy process in a low income country: a qualitative study of stakeholder perceptions of the challenges to effective inclusion in Malawi. BMC Health Serv Res. 2021 Sep 18;21(1):984.
33. Sasie SD, Van Zuylen P, Ayano G, Aragaw FM, Spigt M. Information sharing across institutions: Practices and barriers during public health emergencies in Ethiopia. Int J Med Inform. 2024 Jun;186:105439.
34. World Health Organization. One Health [Internet]. 2025 [cited 2026 Jan 16]. Available from: https://www.who.int/health-topics/one-health

35. Alcayna T, Kellerhaus F, Tremblay L, Fletcher C, Goodermote R, Santos-Vega M, et al. Integrating anticipatory action in disease outbreak preparedness and response in the humanitarian sector. BMJ Glob Health. 2025 Jul 23;10(7):e017721.
36. Dichter JR, Kanter RK, Dries D, Luyckx V, Lim ML, Wilgis J, et al. System-Level Planning, Coordination, and Communication. Chest. 2014 Oct;146(4):e87S-e102S.
37. Kousgaard MB, Scheele CE, Vrangbæk K. Inter-Sectoral Collaboration in Municipal Health Centres: A Multi-Site Qualitative Study of Supporting Organizational Elements and Individual Drivers. Int J Integr Care. 2019 Jun 21;19(2):9.

Annexes

Annex 1: Search strategy

Search number	Query	Results
9	((((multi sectoral OR multi sector OR cross-sector OR intersectoral) OR (intersectoral collaboration[MeSH Terms])) OR (linkage*)) AND ((Public Health emergency OR NPHA OR national emergency OR disaster) OR (disaster management[MeSH Terms]))) AND (barriers OR challenges OR obstacles OR difficulties OR issues)	2,245
8	barriers OR challenges OR obstacles OR difficulties OR issues	3,130,749
7	(Public Health emergency OR NPHA OR national emergency OR disaster) OR (disaster management[MeSH Terms])	921,918
6	disaster management[MeSH Terms]	28,154
5	Public Health emergency OR NPHA OR national emergency OR disaster	921,918

4	((multi sectoral OR multi sector OR cross-sector OR intersectoral) OR (intersectoral collaboration[MeSH Terms])) OR (linkage*)	207,639
3	linkage*	192,545
2	intersectoral collaboration[MeSH Terms]	2,865
1	multisectoral OR multisector OR cross-sector OR intersectoral	12,810

Annex 2: Representation of the Provincial/ District health staff

**Province**	**District**	**Number of KIIs**
Central	Kandy<br>Malate<br>Nuwaraeliya	3
Eastern	Ampara<br>Batticaloa<br>Trincomalee	3
North Central	Anuradhapura<br>Polonnaruwa	2
Northern	Jaffna<br>Kilinochchi<br>Mannar<br>Mullaitive<br>Vavuniya	3

North Western	Kurunegala<br>Puttalam	2
Sabaragamuwa	Kegalle<br>Ratnapura	2
Southern	Galle<br>Matara<br>Hambanthotha	3
Uva	Badulla<br>Monaragala	2
Western	Colombo<br>Gamapaha<br>Kalutara	8

518

Ethics Approval Letter

Click here to access/download
Other
ERC Approval.pdf

Click here to access/download
Other
Revised Manuscript - Clean version - 2026.04.25.docx

Challenges in strengthening multi-sectoral action for optimum preparedness and response for public health emergencies in Sri Lanka

Abstract

Effective coordination between health and other sectors facilitates a better preparedness and response for public health emergencies (PHEs). Sri Lanka is a lower-middle-income island-nation that often contends with multiple PHEs~~public health emergencies~~ such as floods and disease epidemics. In preparing and responding to these PHEs, different units of the Ministry of Health (MOH) address the functions of a typical National Public Health Agency in Sri Lanka. This study was done to describe current approaches to multisectoral action and to explore the challenges to multisectoral action involving the MOH in preparedness for and response to PHEs in Sri Lanka, with particular emphasis on policies, agreements and legislation. The study included a risk assessment, stakeholder-mapping, a desk-review of literature and documents available with institutions which are potential stakeholders as well as qualitative analysis of key informant interviews (KIIs) covering the nine provinces of Sri Lanka. The risk assessment was done to identify potential PHEs. Stakeholder mapping was done to identify key stakeholders ~~while the~~with a stakeholder grid ~~was prepared to identify key stakeholders~~ and to determine their level of engagement. The desk review aimed to identify the deficiencies of the existing major policy documents for multisectoral action. The study team conducted 51 KIIs from April to May 2025 to synthesise challenges using a semi-structured questionnaire with purposively selected team-members involved in different aspects in multisectoral action~~respondents from April to May 2025~~. The US CDC model on public health linkages served as the conceptual framework, including four domains: 1) policies/agreements/legislation, 2) infrastructure, 3) governance/funding/logistics, and 4) people-organization culture. Thematic coding analysis was performed~~ to synthesise challenges under these domains~~. The main challenges imposed by policy deficiencies to multisectoral action ~~involving the MOH~~ included: lack of inclusive engagement of stakeholders in policy formulation leading to fragmented implementation, not having a strong policy architecture for collaboration, non-adaptive policies and vague terminology giving rise to uncertainties of interpretations. Challenges imposed by policy-deficiencies on other three domains included: sub-optimum data management, delayed fund mobilization, lack of mediation of activities of NGOs, less-customized risk communication, and ad-hoc collaborative efforts between stakeholders. To address these challenges, the study recommends adapting existing policy documents, clarifying decentralization and sub-national implementation arrangements, establishing inter-stakeholder operational guidelines, and strengthening intersectoral awareness of roles and communication mechanisms.

Keywords: Public Health Emergencies; multisectoral action; challenges for collaboration; NPHA; policy challenges; policy deficiencies; Sri Lanka

Introduction

Public Health Emergencies (PHEs) encompass acute events – natural or manmade – that challenge health systems due to their scale, timing and unpredictability with routine capacities been overwhelmed (1,2). The COVID-19 pandemic shed light on the importance of rapid and coordinated health system responses to PHEs. PHEs threaten lives and disrupt economies. It is therefore essential to effectively organize health systems to enhance their response capacity and resilience. PHEs pose direct and indirect threats to all building blocks of the health system- service delivery, health workforce, information systems, access to medicines, financing, and leadership/governance (2,3). Multisectoral action is necessary to strengthen all these building blocks. This study examines approaches ~~to~~ and challenges in multisectoral action for enhancing health system preparedness for PHEs, drawing on experiences in Sri Lanka.

Sri Lanka, a lower-middle income country with a population of approximately 22 million is no exception to the impact of PHEs with periodic events like floods, adverse weather conditions, and epidemics including dengue and COVID-19. The World Health Organization (WHO) states that multisectoral actions can strengthen "country ownership, accountability, stewardship of resources, and organizational effectiveness around health emergency preparedness, readiness and response"(4). Further, when multisectoral action is taken during the preparatory phase rather than the response phase, the benefits are greater (4).

Many models or frameworks have been developed to depict the level of preparedness and response for PHEs through multisectoral action (3,5–10). The multisectoral action in the form of preparedness coordination reflects "deliberate collaboration between stakeholders from multiple and diverse sectors and disciplines working towards the shared goal of enhanced health emergency preparedness by leveraging knowledge, expertise, strengths, reach and resources" ~~preparedness coordination framework highlights key elements of such an approach~~ (4). US-CDC has proposed a comprehensive framework to highlight "information on practical approaches public health leaders may consider using to strengthen linkages and their enablers for improved public health impact in their own national contexts" (11). These linkages have been defined as "practical, replicable activities or actions that facilitate collaboration between public health functions or organizations to improve public health impact"(11). In this model, the linkages have been elaboratively categorized under five levels; functional, multifunctional, multisectoral, multilevel and international (11). Four enablers have been mentioned in this model: "policies, agreements and legislation", "infrastructure", "governance, funding and logistics" and "people and organizational culture"(11).

While all these four enabler-domains are important, “policies, agreements and legislation” drive the coordination of multisectoral response in PHEs. Out-of-date policies and lack of understanding on the available policies have been identified as challenges to optimum response in PHEs in the global literature(2). Meanwhile, characteristics of a successful health system include resilience and the capacity to learn from experience, feeding knowledge and data back into the policy (12).

Literature on “policies, agreements and legislation” for multisectoral action for PHEs in Sri Lanka is scarce and has not been consolidated. Thus, the current study aims to describe the current risk of PHEs in Sri Lanka, and to examine the- approaches and challenges of multisectoral action in response efforts-, with particular emphasis on policies, agreements, and legislation.

Methods

The study included a national risk assessment for PHEs, stakeholder mapping, desk review, and a qualitative study covering respondents in all 25 districts and nine provinces of Sri Lanka (13,14).

The risk assessment was done to determine the priority PHE risks presently faced by Sri Lanka, which warrant multisectoral action. This was done with secondary data available at Disaster Preparedness and Response Division, Provincial Directorate of Health Services and Regional Directorate of Health Services for the last 10 years as well as with inputs from the experts in those institutions. The secondary data includeddata included preparedness plans for PHEs and reports available on response phases. The actual frequency of events, likelihood of the event and the impact of the event were judged(15). In addition, the details about the stakeholders and their extent of involvement were asked from the experts in the above institutions.

A stakeholder mapping exercise was conducted to identify the major stakeholders of multisectoral action and assess their degree of engagement in PHEs. The stakeholders who have been involved or who are expected to be involved in potential events were also documented, through a stakeholder mapping activity, following guidance from the WHO “Multisectoral preparedness coordination framework” (4). The details obtained from the experts in the risk assessment was used in this process. Each stakeholder was rated as “high or low” for the two domains of “impact” and “influence” (16); “impact” was defined as “how much of an impact does the stakeholder have on preparedness and response on PHEs” and “influence” was defined as “how much authority does the stakeholder have on preparedness and response on PHEs” (16)Those with high levels of both were identified. Level of engagement was categorized as: consult (high interest- high influence), convince (high influence- low interest), update (high interest- low influence) and monitor (low influence- low interest) (16)Co-authors discussed these assignments for consensus, and where needed, also consulted the technical experts in the institutionsrelevant officers who are related to

multisectoral actions. Participants for KIIs were recruited from the institutions identified as having "consult" and "convince" levels of engagement (16).

A desk review was conducted to collate evidence for facilitators and barriers for multisectoral action and its governance. This included an online search in PubMed and Google Scholar and a review of reports on preparedness planning and responses from the various stakeholders. Expert feedback provided recommendations for additional grey literature and policy documents to include for review. In PubMed, subject heading and keyword searches were done. The subject heading search was done by exploring the relevant MeSH terms and keywords. The complete search strategy is shown in Annex 1. Advance search option was used in Google Scholar. The reports that were reviewed included documents related to PHE preparedness and response activities available with stakeholders.

Interviews were conducted with stakeholders with experience working in the institutions that were determined to have both high impact and influence in multisectoral action (17–24). Altogether, the team conducted 51 KIIs between April and May 2025 following recommended methodology(22–24) ~~(16–20)~~. Purposive sampling was done by discussing with staff members of selected institutions. Participants included different team members involved in: policy planning, implementation, research, administration, training, and financing. They were reached out with consultations with the Heads of Institutions, Furthermore, it was ensured that the participants covered both government sector and non-governmental sectors, such as the Sri Lanka Red Cross. In the government sector, stakeholders from national, provincial, district and divisional levels were included. There are 9 Provincial Director of Health Services (PDHS) and 26 Regional Director of Health Services (RDHS offices) in Sri Lanka, hence more participants were from those institutions. Both curative and preventive sectors as well as health and non-health sectors were covered. The institutional breakdown of the 51 KIIs conducted is shown below in Table 1.

Table 1: KIIs conducted in different institutions

Type of institution	Number of KIIs
Line Ministry health institutions	05
Offices of Provincial and Regional Directors of Health Services (PDHS & RDHS)	28
Offices of Medical Officer of Health (MOH)	04
Curative health institutions	02
Disaster Management Centre	03
Provincial Council, District Secretariat, Divisional Secretariat	04

Security forces	02
Academia	01
NGOs	02
Total	51

Representation of the Provincial/ District health staff in the KIIs is shown in Annex 2.

~~Purposive sampling was done by discussing with staff members of selected institutions.~~ A semi-structured questionnaire was prepared. The contents of the questionnaire were based on the four-domains of the US CDC framework (annex 3). In this model, four enablers of public health linkages have been proposed as: 1). Policies, agreements and legislation, 2). Infrastructure, 3). Governance, funding and resources and 4). People and organizational culture. Interviews were done by study investigators with one being the moderator and another being a note taker. The average duration of a KII was 20 minutes (range 15 to 40 minutes). Interviews were audio-recorded with written consent given by participants. The audio recordings were transcribed following principles from Baily et al (2008) by two authors independently and spot-checked for quality. Data were analysed and interpreted concurrently using a convergent-parallel design to integrate findings across methods(25). Thematic analysis was done using the deductive approach under the four domains of the US CDC framework (26).

Ethics approval was obtained from the Ethics Review Committee of National Institute of Health Sciences (NIHS/ERC/24/77R).

Results

The results are presented in the order of: 1. risk assessment and stakeholder mapping, 2. desk review findings including the current structure of delivery of NPHA functions in Sri Lanka, and 3. findings from the KIIs.

Actual and potential PHEs of Sri Lanka and stakeholder-grid

The risk assessment revealed that PHEs in the previous 10 years included: COVID-19, dengue outbreaks, floods, landslides, explosions, and events due to adverse weather conditions. In addition, potential future event risks include: tsunami, other disease epidemics, chemical accidents, and recurrences of those that have been reported within last 10 years. The stakeholder-grid that was prepared to identify the organizations from which to draw the KII respondents is shown below in Table 2.

Table 2: Stakeholder grid

Organization	Type of influence	Level of impact	Level of influence	Level of engagement

Disaster Management Centre	-Policy formulation<br>-Administration<br>-Coordination	High	High	Consult
Disaster Preparedness and Response Unit- Line Ministry	-Policy formulation<br>-Coordination	High	High	Consult
Offices of Provincial Director of Health Services	-Implementation<br>-Technical	High	High	Consult
Offices of Regional Director of Health Services	-Implementation<br>-Technical	High	High	Consult
Epidemiology Unit	-Policy formulation<br>-Technical	High	High	Consult
Family Health Bureau	-Policy formulation<br>-Technical	High	High	Consult
Health Promotion Bureau	-Policy formulation<br>-Technical	High	High	Consult
Environmental & Occupational Health Unit	-Policy formulation<br>-Technical	High	High	Consult
National Dengue Control Unit	-Policy formulation<br>-Technical	High	High	Consult
Anti Malaria Campaign	-Policy formulation<br>-Technical	High	High	Consult
Provincial Councils	-Administration<br>-Implementation<br>-Financial	High	High	Consult
District Secretariate	-Administration<br>-Implementation<br>-Financial	High	High	Consult
Divisional Secretariate	-Administration<br>-Implementation	High	High	Consult
Offices of Medical Officers of Health	-Implementation<br>-Technical	High	High	Consult
Curative institutions	-Implementation<br>-Supportive	High	High	Consult

Security forces	-Supportive<br>-Implementation	High	High	Consult
Non-Governmental Organizations	-Supportive<br>-Financial	High	Low	Convince
Universities	-Technical	High	Low	Convince

Except for the NGOs and universities, all other institutions mentioned in the above table were categorized as having a high level of impact and influence and thus being in the "consult" level of engagement. The former two were assigned the "convince" level of engagement(16).

Policy documents

Formatted: Font: Bold

From the desk review and the discussions with experts in the institutions, eight policy documents related to multisectoral action were identified. These eight policy documents were categorized as having: 1) general components of disasters, 2) health-related disasters as the main focus, 3) risk communication, and 4) security as the main focus. An analysis of the policy documents related to multisectoral actions in PHEs and their gaps are shown in Table 3 with a summary of each provided below.

The Disaster Management Act gave the opportunity to form the Disaster Management Centre (DMC) which is the lead agency for coordinating preparedness, mitigation, and recovery efforts in emergencies/disasters(27). The National Disaster Management Plan (NDMP) 2023-2030 serves as Sri Lanka's comprehensive framework for disaster risk management(28). It encompasses various hazards, including health emergencies such as epidemics and pandemics. The National Emergency Operation Plan (NEOP), provides a comprehensive framework for coordinating emergency responses across various hazards aiming to enhance national resilience through multi-sectoral coordination, early warning systems, and standardized response protocols(29). The Sri Lanka National Health Policy (2016-2025) is built upon the concept of "Universal Health Coverage" (UHC), aiming to ensure accessibility to quality services with financial protection. The National Health Strategic Master Plan 2016–2025 underscores a commitment to addressing emerging health challenges, improving infrastructure, and ensuring compliance with international health standards, including the IHR 2005(30). The National Risk Communication Strategic Plan of Sri Lanka aims to strengthen Sri Lanka's capacity to provide accurate information, counter misinformation, and build public trust in health interventions. The Public Security Ordinance No. 25 of 1947 is a key legal instrument in Sri Lanka that enables the integration of public health and security authorities during emergencies, including pandemics and disease outbreaks(31). Although the primary mandate of Sri Lanka Police Ordinance No. 16 of 1865 is on law enforcement and community policing, its provisions offer potential leverage points for extending the role of the police force in PHEs(32).

Table 3: **Existing policy documents and their gaps**

Policy document	Deficiencies
**Policies related to general components of disasters as the main focus**	
1.Disaster Management Act No. 13 of 2005	Designed mainly for natural disasters and not for disease epidemics and pandemics.<br>Coordination on preparedness and response of DMC based on disease surveillance is not optimal with health and animal sector.
2. National Disaster Management Plan, 2023-2030	No policy mandate for real time integrated Disease Surveillance System available, which hinders early risk mitigation and prevent health emergencies.
3. National Emergency Operation Plan (NEOP)	IHR requires detailed capacities in surveillance and laboratory diagnostics. The NEOP lacks explicit references to national laboratory networks or protocols for epidemiological surveillance.
**Policies related to disasters with health as the main focus**	
4. National Health Policy 2016 – 2025	Institutionalization of multisector coordination is weak in the policy text. While the policy endorses intersectoral action, it stops short of prescribing statutory or permanent governance arrangements such as having district multisector fora with defined mandates and reporting.<br><br>Subnational operational capacity and decentralisation not fully detailed - the Policy's national orientation needs clearer provisions for district/divisional roles and resourcing.
5. National Health Strategic Master Plan 2016 – 2025	Human Resources for Health (HRH) strategies exist but implementation gaps like uneven distribution, retention problems in rural/estate areas, unclear task-shifting policies persist. Strengthening HRH was repeatedly identified as a constraint.<br><br>The plan references eHealth but interoperability and nationwide data governance were not fully implemented. This created fragmented records and poor information flows.<br><br>The Master Plan recognises the private sector but lacks a strong, operational regulatory framework and clear engagement models to align private provision with public goals.
**Policies related to risk communication**	

6. National Risk Communication Strategic Plan for Public Health Emergencies in Sri Lanka	Although the plan emphasizes community engagement, there is little indication of tailored messaging for different socio-cultural groups like urban slum dwellers and estate communities beyond general language translation.
**Policies related to security**	
7.Public Security Ordinance	There is no dedicated mechanism to ensure routine collaboration between the Ministry of Health and the Ministry of Defense for health emergency readiness.
8.Sri Lanka Police Ordinance	The Police Ordinance does not explicitly include public health as an area of responsibility, leading to potential legal uncertainties when police are called upon for health-related duties.

Current structure of delivery of NPHA functions in Sri Lanka

Following the desk review and discussions with the institutions, the current mechanisms of delivering NPHA functions were summarized. Currently, there is no single entity named as a National Public Health Agency (NPHA) in Sri Lanka. The central Ministry of Health issues guidelines while the provincial, district, and divisional level stakeholders contribute to the implementation. At the central level, the Quarantine Unit and Epidemiology Unit function as co-national focal points under the International Health Regulations IHR 2005 while the latter leads in disease surveillance as well. Health sector preparedness and response in general are coordinated by the Disaster Preparedness and Response Division of Ministry of Health. There are other specific control programs like the National Dengue Control Unit and the National Tuberculosis and Chest Diseases Control Programme. The non-health stakeholders like the Provincial Councils, Local Government bodies, District Secretariates and Divisional Secretariates play critical coordination and response-implementation roles in the preparedness and response in PHEs.

Building on the limitations identified in the desk review, challenges to multisectoral action as suggested by the key informants are shown below in Table 4. The experts pointed out eight challenges in relation to policy-agreement-legislation domain, highlighting deficiencies in policy focus, priorities, architecture, implementation, and monitoring. The second domain that covered physical and digital infrastructure yielded seven challenges that covered aspects like lack of common physical infrastructure as well as sub-optimal data management. Challenges in relation to fund mobilization, donation processes, and NGO coordination were highlighted for the domain on "governance-funding and resources". Six challenges that covered aspects like training fora,

scopes of work, standard operating protocols, sustainability, and integration of social media were described under the domain on "people-organization culture".

Table 4: Challenges as suggested by the Key Informants

**Policies- agreements-legislation domain**	**Infrastructure domain**
• Major policy and legislative instruments emphasize response actions more than preparedness<br>• Priorities are not aligned across sectors in relation to PHEs<br>• No strong policy architecture is present for collaboration between actors<br>• NGOs have to fulfil their own funders' requirements<br>• It is often necessary to go through default hierarchies of the administration in incident commanding system, limiting efficiency<br>• There is a lack of documented policies on data sharing<br>• There is a lack of monitoring mechanisms for multisectoral action for PHEs<br>• There is a lack of policies on documentation in relation to preparedness and response for PHEs	• Limited identification of a common infrastructure for the response phase was noted<br>• Non-availability of an integrated laboratory network was found<br>• There is a lack of resource mapping across different sectors<br>• Fragmented data gathering is observed leading to duplication of efforts<br>• There is no integrated database<br>• Discrepancies between data collected from different sources are noted<br>• Minimum use of digital technology in data management is seen, leading to more manual efforts
**Governance, funding and resources domain**	**People and organization culture domain**
• Non-availability of a separate fund to mobilize in PHEs was noted<br>• Undue delays in fund mobilization are experienced<br>• There is no mechanism to jointly monitor the functions and processes related to PHEs<br>• Delays in mobilizing and redistribution of resources in the response phase are noted<br>• Duplication is found in activities done by NGOs in some instances<br>• There is lack of clarity on donation processes	• No common training fora are found<br>• Lack of awareness on others' scope in PHEs was noted<br>• Lack of clarity on SOPs on other stakeholders was noted<br>• Coordination often depends on personal connections that may not sustain with changes in human resources<br>• Non-health focal points sometimes lack awareness on and knowledge of health system operations and needs

	• Non-availability of a system to use the beneficial effects and to reduce harmful influence of social-media was found

By collating the evidence from the literature review, review of reports~~desk review~~ and KIIs, the main challenges in relation to policy deficiencies were synthesized. A key challenge identified was the lack of inclusive engagement of stakeholders in policy formulation, which has led to fragmented implementation. This challenge is further exacerbated by the absence of a strong policy architecture to support collaboration between actors and to enable timely adaptation to meet changing needs. As a result, outdated and uncoordinated policies create uncertainty about how provisions apply, particularly within a decentralized system, and contribute to inefficiencies in administrative, operational, and financial processes during PHEs. Another challenge identified is the absence of policy provisions for a common database, limiting the opportunities for data integration across sectors. The lack of an established laboratory network represents a manifestation of this gap. Additionally, vague terminology within security polices was highlighted as another challenge as it gives rise to uncertainties in interpretations.

Discussion

This is the first documented ~~qualitative~~ study done in Sri Lanka at the national level on approaches and challenges for effective multisectoral action in PHEs. The findings revealed current PHE risks, identified key stakeholders for multisectoral action in PHEs in Sri Lanka, and explored potential challenges in relation to the four domains of the US CDC model: policies/ agreements/legislations, infrastructure, governance/funding/logistics, and people/ organizational culture. Our stakeholder mapping exercise identified a large number (n=16) of categories of stakeholder institutions that were considered “high” in terms of their influence and impact, and in totality, represent a complex environment of stakeholders that require coordination and consultation. The findings suggest that there is no uniform framework that units are using for multisectoral governance.

Several methodological strengths which are potentially applicable in other settings too, can be noted. The stakeholders and their level of engagement were identified with the inputs of experts who actually involve in multisectoral activities within related institutions. KIIs were done covering all types of stakeholders with high influence and impact. These two steps have enabled the inclusivity. The synthesis of the challenges according to US CDC model ensured the comprehensiveness of findings as well as enabled their potential comparison with other settings.

Under “policies, agreements, and legislation”, the major challenges identified included policy deficiencies related to policy architecture, policy inclusiveness, policy adaptation, and vague

policy terminology. These deficiencies are interconnected and contribute to the root cause of many other challenges in achieving multisectoral action for health. For example, non-adaptive and out-of-date policies hinder optimal functioning and efficient processes within decentralized governance structures. In addition, vague terminologies in legal policy documents, particularly around security-related policies, create uncertainties in interpretation and constrains their application in supporting PHE response efforts. Emphasizing the importance of clarifying legal provisions, the National Summit on Public Health Legal Preparedness in 2007 highlighted the need for ensuring legal preparedness in the context of PHEs (24). Limited enforceability, duplication of efforts, fragmented involvement, vague mandates in inter-ministerial level are several ~~Many of the other policy-rela~~policy-related ~~ted~~ challenges identified in global literature which are ~~this study are~~ aligned with those elicited in the present study ~~have been reported globally as well as in regional literature, including in a comparative study done across nine countries~~ (33–37).The reported literature shows the deficiencies of the health policy processes hindering aspects like inclusiveness and meaningful engagement in PHEs (33–37).

The identified policy deficiencies lead to difficulties in relation to other domains of the US CDC framework as well. One example is fragmented coordination of the utilization of digital infrastructure (i.e. the infrastructure domain) leading to duplications in data management. For example, in a flood event, data are collected by the different units of the Ministry of Health itself using different data templates that creates unnecessary workloads ~~for ground~~for ground-level staff. This was again apparent in the response events related to Ditwah cyclone in late 2025 as well(38). In epidemic events, identified deficiencies give rise to undue delays in funding mobilization that include transferring of funds along traditional hierarchical levels rather than having a more agile system in place as part of preparedness activities. In general, the absence of policy provisions to mandate and enable multisector collaboration and coordination, shared preparedness training platforms, and a common understanding of roles, scope of work, and resources across sectors, contributes to inefficiencies in response efforts. For example, health units may be unable to leverage support from non-health stakeholders during the response phase if roles and responsibilities are not clearly understood. Hence addressing the policy domain as a primary driver for multisectoral action, serves to intervene in challenges under other domains as well.

The importance of policy provisions is further highlighted by the absence of a provision to have a structural coordinating agency like an NPHA. Coordinating agencies are particularly critical as poor stakeholder engagement leads to fragmentation of services in relation to preparedness and response to PHEs(36). Even though the services related to PHEs are delivered from different units within the Ministry of Health, this fragmentation leads to duplication and gaps in some control measures. Similarly, the lack of coherent implementation and central monitoring lead to misalignment of priorities across organizations.

Under infrastructure, the major challenges identified in the study were non-identification of common physical infrastructure and sub-optimal data management. Exacerbated by the absence of an integrated real-time database and lack of documented policies on data sharing, this creates a vicious cycle leading to poor data integration and management. This challenge was also identified in a mixed methods study in Ethiopia where limited digital infrastructure and incompatibility of data formats were identified as barriers to effective data coordination (37). Sri Lanka should develop integrated unified databases to be used in PHEs to integrate operational data. Currently such databases – linking curative and preventive efforts – are found in relation to communicable conditions like dengue and tuberculosis . Once developed, such a platform can integrate health surveillance data with data from other sectors facilitating initiatives like the One Health approach (39). The absence of a laboratory network, which was highlighted as another challenge, can also be addressed by a unified database and real-time data exchange. Thus, the main national laboratory should be linked with those at peripheral levels and this network could be incorporated to the integrated database with surveillance data.

For the "governance-funding and resources" domain, several financial difficulties were highlighted. Given this context, lack of an anticipatory pre-arranged financing mechanism makes the country more vulnerable(40). Outdated financial procedures should be reviewed and policy adaptations undertaken. Under the domain of people and organization culture, it was elicited that most connections between organizations are on a personal level and not due to system-driven mechanisms, policies, or practices. In contrast, the better approach is to have well-laid system-level planning which is more sustainable(41). A multi-site qualitative study has shown that, although ad-hoc interactions between professions may provide some benefit, it would not give rise to "systematic forms of collaboration"(42). The involvement of NGOs yield potential benefits, however ensuring non-duplication and balancing the commercial interests become challenges. As an example in the Ditwah response period, several NGOs were interested in arranging medical camps, however the funders' interests made potential duplications. In some settings, the same geographical area was targeted due to funders' interests. Balancing the commercial interests with health policies, like in the case of child milk-powder promotion, needed extra efforts.

Formatted: Font: (Default) Times New Roman, 12 pt

The WHO has emphasized the value of proactive considerations and far-sighted policies over limited response measures (4). Within this context, the way forward must include several considerations as shown in Table 5.

Table 5: Recommendations in addressing the challenges

Core-challenges	Key recommendations
Absence of a strong policy architecture to support collaboration between actors and to enable timely adaptation to meet changing needs	Policy adaptations with timely revision of currently existing policy documents

Formatted Table

Inefficiencies in administrative, operational, and financial processes during PHEs specially within the decentralization	Defining clearer terms in relation to decentralization and sub-national implementation
Lack of inclusive engagement of stakeholders in policy formulation and fragmented implementation	Better sectoral dialogues
Absence of policy provisions for a common database	Raising intersectoral awareness on mutual scopes
Vague terminology within security polices	Having specific inter-stakeholder operational guidelines

Formatted Table

Policy adaptations with timely revision of currently existing policy documents are one such worthy endeavour. Within this, defining clearer terms in relation to decentralization and sub-national implementation are recommended. Having specific inter-stakeholder operational guidelines, raising intersectoral awareness on mutual scopes, and better sectoral dialogues are other key considerations. Once the policy domain is addressed, it will facilitate addressing the challenges of the other three domains as well. Challenges like the presence of data discrepancies, fragmented data gathering, absence of a common database can be solved once the data sharing provisions are thus streamlined through policies . At the same time, the funding related challenges mentioned under domain three can also be addressed and the human resource related challenges such as training and raising the awareness will also be facilitated once the policies are clearer with the policy reforms.

There were several limitations of the study. The challenges identified in the study were based on the subjective experiences and opinions of individuals and may be informed by one event or limited to one setting. To mitigate the potential for bias, a larger number of KIIs across a range of organizations – identified in the stakeholder mapping exercise – were conducted to ensure the inclusion of diverse perspectives and to strengthen the robustness of emergent themes. ~~Furthermore a consistent prioritization technique was used that helped standardize inputs more representative.~~

Conclusions and recommendations

This novel study identified critical challenges with multisectoral collaboration in Sri Lanka, involving policy, infrastructure, funding, and people organization culture. We found that services related to PHEs are delivered by different units in Sri Lanka without the existence of an NPHA entity to support coordination. Policy deficiencies give rise to challenges including lack of inclusive engagement of stakeholders in policy formulation leading to fragmented implementation, absence of a strong policy architecture for collaboration, lack of policy adaptation and vague terminology giving rise to uncertainties in interpretations. In addition, policy deficiencies impose

challenges for the other three domains of the US CDC framework as well. These include: sub-optimal data management, delayed fund mobilization, suboptimal coordination of NGOs, and ad-hoc collaborative efforts between stakeholders. To address these challenges, policy adaptation of existing policy documents, defining clearer terms in relation to decentralization and sub-national implementation (i.e. clarifying roles, authority, and responsibilities), having specific inter-stakeholder operational guidelines, raising intersectoral awareness on mutual scopes and system-driven intersectoral communication are recommended as a first step toward a truly multisectoral approach to preparedness and response to PHEs in Sri Lanka.

References

1. Nelson C, Lurie N, Wasserman J, Zakowski S. Conceptualizing and defining public health emergency preparedness. Am J Public Health. 2007 Apr;97 Suppl 1(Suppl 1):S9-11. doi:10.2105/AJPH.2007.114496 PubMed PMID: 17413078.
2. Khatri RB, Endalamaw A, Erku D, Wolka E, Nigatu F, Zewdie A, et al. Preparedness, impacts, and responses of public health emergencies towards health security: qualitative synthesis of evidence. Archives of Public Health. 2023 Nov 30;81(1):208. doi:10.1186/s13690-023-01223-y
3. Olu O. Resilient Health System As Conceptual Framework for Strengthening Public Health Disaster Risk Management: An African Viewpoint. Front Public Health. 2017 Sep 28;5. doi:10.3389/fpubh.2017.00263
4. World Health Organization. Multisectoral Preparedness Coordination Framework: best practices, case studies and key elements of advancing multisectoral coordination for health emergency preparedness and health security. Geneva; 2020.
5. Ario AR, Makumbi I, Bulage L, Kyazze S, Kayiwa J, Wetaka MM, et al. The logic model for Uganda's health sector preparedness for public health threats and emergencies. Glob Health Action. 2019;12(1). doi:10.1080/16549716.2019.1664103
6. Hong Y, Zhao J, Zhang Y, Su Q. Motivating public health emergency preparedness cooperative behaviors: based on the expectancy disconfirmation model. J Health Organ Manag. 2023;37(6–7). doi:10.1108/JHOM-09-2022-0268

Formatted: Indent: First line: 0", Tab stops: 0.5", Left

7. Lowe AE, Kraft C, Kortepeter MG, Hansen KF, Sanger K, Johnson A, et al. Developing a Rapid Response Single IRB Model for Conducting Research during a Public Health Emergency. Health Security. 2022. doi:10.1089/hs.2021.0181

8. Stoto MA, Nelson C, Savoia E, Ljungqvist I, Ciotti M. A Public Health Preparedness Logic Model: Assessing Preparedness for Cross-border Threats in the European Region. Health Secur. 2017;15(5). doi:10.1089/hs.2016.0126

9. Eerens D, Hrzic R, Clemens T. The architecture of the European Union's pandemic preparedness and response policy framework. Eur J Public Health. 2023;33(1). doi:10.1093/eurpub/ckac154

10. Yaylali E, Ivy JS, Taheri J. Systems engineering methods for enhancing the value stream in public health preparedness: The role of Markov models, simulation, and optimization. Public Health Reports. 2014;129. doi:10.1177/00333549141296S419

11. United States Centers for Disease Control and Prevention. Improving Linkages between Public Health Functions and Organizations- A framework for National Public Health Institutes. 2024.

12. Mills A. Health Care Systems in Low- and Middle-Income Countries. New England Journal of Medicine. 2014 Feb 6;370(6):552–7. doi:10.1056/NEJMra1110897

13. Abusayeed MH, Rampatige RGV, Peiris SL, Galappathi H. A Qualitative Assessment of the Health Information System in Sri Lanka using HMN assessment tool. Sri Lanka Journal of Bio-Medical Informatics. 2010;1(2). doi:10.4038/sljbmi.v1i2.1664

14. Prakash SL, Abeykoon P, Niwarthana G, Malshan B, Nanayakkara S, Nanayakkara J, et al. Community participating 'smoke free zones' against second hand smoking in Sri Lanka; present and future. Tob Induc Dis. 2021;19(1). doi:10.18332/tid/141029

15. Alharbi NK, Alariqi L, Mantero J, Zeyad L, Mercy K, Xiang N, et al. Methods and tools for rapid risk assessments for acute public health emergencies. J Infect Public Health. 2025 Dec;18(12):102965. doi:10.1016/j.jiph.2025.102965

16. World Health Organization. Stakeholder mapping and engagement-WHO SEARO Salt Reduction Toolkit.

17. Zacharia B, Pai PK, Paul M. Focus Group Discussion as a Tool to Assess Patient-Based Outcomes, Practical Tips for Conducting Focus Group Discussion for Medical Students—Learning With an Example. J Patient Exp. 2021;8. doi:10.1177/23743735211034276

18. Khan NA, Abedin S. Focus Group Discussion. In: Principles of Social Research Methodology. 2022. doi:10.1007/978-981-19-5441-2_26

19. Ahmed SK. The pillars of trustworthiness in qualitative research. Journal of Medicine, Surgery, and Public Health. 2024;2. doi:10.1016/j.glmedi.2024.100051

20. Adler RH. Trustworthiness in Qualitative Research. Journal of Human Lactation. 2022;38(4). doi:10.1177/08903344221116620

21. Kakar ZUH, Rasheed R, Rashid A, Akhter S. CRITERIA FOR ASSESSING AND ENSURING THE TRUSTWORTHINESS IN QUALITATIVE RESEARCH. International Journal of Business Reflections. 2023;4(2). doi:10.56249/ijbr.03.01.44

22. Lokot M. Whose Voices? Whose Knowledge? A Feminist Analysis of the Value of Key Informant Interviews. Int J Qual Methods. 2021 Jan 1;20. doi:10.1177/1609406920948775

23. Pahwa M, Cavanagh A, Vanstone M. Key Informants in Applied Qualitative Health Research. Qual Health Res. 2023 Dec 30;33(14):1251–61. doi:10.1177/10497323231198796

24. Luetke Lanfer H, Krawiec S, Schierenbeck M, Touzel V, Reifegerste D. Balancing between reality, ideality, and equity: critical reflections from recruiting key informants for qualitative health research. BMC Med Res Methodol. 2024 Nov 12;24(1):276. doi:10.1186/s12874-024-02403-2

25. Ozawa S, Pongpirul K. 10 best resources on … mixed methods research in health systems. Health Policy Plan. 2014 May 1;29(3):323–7. doi:10.1093/heapol/czt019

26. Bingham AJ. From Data Management to Actionable Findings: A Five-Phase Process of Qualitative Data Analysis. Int J Qual Methods. 2023 Jan 10;22. doi:10.1177/16094069231183620

27. Disaster Managemnet Centre. Disaster Management Act No.13 of 2005 [Internet]. 2005 [cited 2026 Jan 16]. Available from: https://www.dmc.gov.lk/images/DM_Act_English.pdf

28. Disaster Management Centre. The National Disaster Management Plan (NDMP) 2023-2030 [Internet]. 2023 [cited 2026 Jan 16]. Available from: https://www.dmc.gov.lk/images/pdfs/NDMP_V2_withCoverV2.pdf

29. Disaster Management Centre. National Emergency Operation Plan [Internet]. 2017 [cited 2026 Jan 16]. Available from: https://www.dmc.gov.lk/images/pdfs/NEOP/NEOP_English.pdf

30. Ministry of Health Sri Lanka. National Health Strategic Master Plan [Internet]. 2016 [cited 2026 Jan 16]. Available from: https://www.health.gov.lk/moh-pages/health-master-plan-2016-2025/

31. Government of Sri Lanka. Public Security Ordinance [Internet]. 1947 [cited 2026 Jan 16]. Available from: https://www.lawnet.gov.lk/wp-content/uploads/leg_enact_1981/1981Y3V51C.html

32. Government of Sri Lanka. Police Ordinance [Internet]. 1865 [cited 2026 Jan 16]. Available from: https://www.vertic.org/media/National%20Legislation/Sri%20Lanka/LK_Police_Ordinance.pdf

33. Shah I, Elahi N, Alam A, Dawar S, Dogar AA. Institutional arrangement for disaster risk management: Evidence from Pakistan. International Journal of Disaster Risk Reduction. 2020 Dec;51:101784. doi:10.1016/j.ijdrr.2020.101784

34. El-Jardali F, Fadlallah R, Daher N. Multi-sectoral collaborations in selected countries of the Eastern Mediterranean region: assessment, enablers and missed opportunities from the COVID-19 pandemic response. Health Res Policy Syst. 2024 Jan 24;22(1):14. doi:10.1186/s12961-023-01098-z PubMed PMID: 38267995.

35. Miqdadi R, Hamdan M. Key stakeholders’ perspectives of public health emergency preparedness in Palestine: A qualitative exploratory study. Public Health. 2024 Dec;237:232–7. doi:10.1016/j.puhe.2024.10.013 PubMed PMID: 39454233.

36. Masefield SC, Msosa A, Chinguwo FK, Grugel J. Stakeholder engagement in the health policy process in a low income country: a qualitative study of stakeholder perceptions of the challenges to effective inclusion in Malawi. BMC Health Serv Res. 2021 Sep 18;21(1):984. doi:10.1186/s12913-021-07016-9

37. Sasie SD, Van Zuylen P, Ayano G, Aragaw FM, Spigt M. Information sharing across institutions: Practices and barriers during public health emergencies in Ethiopia. Int J Med Inform. 2024 Jun;186:105439. doi:10.1016/j.ijmedinf.2024.105439 PubMed PMID: 38564958.

38. World Health Organization. Cyclone Ditwah 2025 [Internet]. 2026 [cited 2026 Apr 24]. Available from: https://www.who.int/srilanka/emergencies/cyclone-ditwah-2025

39. World Health Organization. One Health [Internet]. 2025 [cited 2026 Jan 16]. Available from: https://www.who.int/health-topics/one-health

40. Alcayna T, Kellerhaus F, Tremblay L, Fletcher C, Goodermote R, Santos-Vega M, et al. Integrating anticipatory action in disease outbreak preparedness and response in the humanitarian sector. BMJ Glob Health. 2025 Jul 23;10(7):e017721. doi:10.1136/bmjgh-2024-017721

41. Dichter JR, Kanter RK, Dries D, Luyckx V, Lim ML, Wilgis J, et al. System-Level Planning, Coordination, and Communication. Chest. 2014 Oct;146(4):e87S-e102S. doi:10.1378/chest.14-0738

42. Kousgaard MB, Scheele CE, Vrangbæk K. Inter-Sectoral Collaboration in Municipal Health Centres: A Multi-Site Qualitative Study of Supporting Organizational Elements and Individual Drivers. Int J Integr Care. 2019 Jun 21;19(2):9. doi:10.5334/ijic.4196 PubMed PMID: 31244563.

Formatted: Indent: Left: 2.22"

Annexes

Annex 1: Search strategy

Search number	Query	Results
9	((((multi sectoral OR multi sector OR cross-sector OR intersectoral) OR (intersectoral collaboration[MeSH Terms])) OR (linkage*)) AND ((Public Health emergency OR NPHA OR national emergency OR disaster) OR (disaster management[MeSH Terms]))) AND (barriers OR challenges OR obstacles OR difficulties OR issues)	2,245
8	barriers OR challenges OR obstacles OR difficulties OR issues	3,130,749
7	(Public Health emergency OR NPHA OR national emergency OR disaster) OR (disaster management[MeSH Terms])	921,918
6	disaster management[MeSH Terms]	28,154
5	Public Health emergency OR NPHA OR national emergency OR disaster	921,918
4	((multi sectoral OR multi sector OR cross-sector OR intersectoral) OR (intersectoral collaboration[MeSH Terms])) OR (linkage*)	207,639
3	linkage*	192,545
2	intersectoral collaboration[MeSH Terms]	2,865
1	multisectoral OR multisector OR cross-sector OR intersectoral	12,810

Annex 2: Representation of the Provincial/ District health staff

**Province**	**District**	**Number of KIIs**
Central	Kandy<br>Matale<br>Nuwaraeliya	3
Eastern	Ampara<br>Batticaloa<br>Trincomalee	3
North Central	Anuradhapura<br>Polonnaruwa	2
Northern	Jaffna<br>Kilinochchi	3

	Mannar<br>Mullaitive<br>Vavuniya	
North Western	Kurunegala<br>Puttalam	2
Sabaragamuwa	Kegalle<br>Ratnapura	2
Southern	Galle<br>Matara<br>Hambantota	3
Uva	Badulla<br>Monaragala	2
Western	Colombo<br>Gampaha<br>Kalutara	8

Annex 3

Semi structured questionnaire

Questions and prompts:

1. What kind of public health emergencies does your institution deal with?

Probe for:

- What have been already dealt with in the last ten years
- What can be anticipated in the coming ten years

2. What can you say about the preparedness of your institution for public health emergencies?

3. Can you explain how does your institution engage in multisectoral collaboration in preparedness and response to public health emergencies?

3. What challenges do you perceive in multisectoral collaboration and its governance in relation to policies, agreements and legislation

Probe for

- Legislative mandates
- Policies governing national– subnational relationships
- Rules and SOPs
- Data-sharing policies and MOUs

4. What challenges do you perceive in multisectoral collaboration and its governance in relation to infrastructure

Probe for:

- ❖ Physical structures for collaboration
- ❖ Public health observatories
- ❖ Information systems optimized for data sharing
- ❖ Conceptual or organizational frameworks for integration
- ❖ Regional public health assets

5. What challenges do you perceive in multisectoral collaboration and its governance in relation to governance, funding, and resources

Probe for:

- ❖ Political support for linkages
- ❖ Funding and resources to support subnational activities,
- ❖ Participation of subnational authorities in planning and implementation lifecycle
- ❖ Effective leadership and management of linkages
- ❖ Aligned or shared goals,
- ❖ Strategic plans,
- ❖ Work plans

6. What challenges do you perceive in multisectoral collaboration and its governance in relation to people and organizational culture

Probe for:

- ❖ Inclusivity of teams
- ❖ Trust
- ❖ Relationships and history of collaboration
- ❖ Effective internal communication
- ❖ Shared nomenclature
- ❖ Investing in skills and professional

7. What else have you got to say about preparing and responding to public health emergencies as far as your institution is concerned?

Thank you for your participation!

Response to the comments of reviewers

Editor

Comment	Actions taken	Section in revised manuscript
Thank you for conducting this timely and impactful research - which will be highly relevant given the current context of Sri Lanka trying to strengthen disaster response and recovery - also will have strong relevance for the future as climate crisis induced PHEs will increase. I agree with the reviewers that clarity in methods and overall coherence could strengthen this paper. Please pay close attention to Reviewer 2 comments on strengthening the methods and coherence of the paper. This is an important paper for policy - and strengthening the methods and coherence will improve the impact the paper can have and academic credibility in terms of research rigour.	Thanks for the comment. All comments of the both reviewers have been addressed as shown below. Close attention was paid to the comments of reviewer 2 on strengthening the methods and the coherence of the paper.	Throughout the manuscript
Additionally, if possible, I also suggest strengthening the discussion section by bringing in the recent cyclone Ditwah into the discussion section. Although, this is a natural disaster at first, as we know natural disasters invariable have an impact on public health (and fall within the definition provided). Including the recent natural disaster in the discussion section and where necessary in conclusion/recommendation will increase the relevance of this paper and improve the uptake in national, regional and global settings. Furthermore, as NGO coordination and other challenges are mentioned and given the role NGOs play in disaster responses - would be good to discuss NGO role and pathways for strengthening that role.	Thanks for the comment. The discussion was made enriched with references to the Ditwah as well as on NGO role.	Discussion section

Reviewer #1:

Comment	Action/s taken	Section in revised manuscript
I commend the authors for doing a deep-dive in addressing a relevant and timely topic. Overall, I liked the mixed method approach involving risk assessment, stakeholder mapping, desk review and hey informant interviews which I thought was quite comprehensive	Thanks for the encouraging comment	NA
The KII interviews:<br><br>The authors were also from the Ministry of Health Sri Lanka which creates a bias that is not addressed in the manuscript.<br><br>It would be better if more details about the interviews (duration, language) were explicitly mentioned. Also mention whether the transcribing was done by two authors independently which would remove biases. The questionnaire should be made available. It would strengthen the study if direct quotes from the interviewees were also included.	Thanks for the comment. The necessary details have been added. The questionnaire has been made available as Annex 4.	Methods section
Desk review:<br><br>I recommend that the authors integrate the desk review findings and KII interview findings instead of presenting them sequentially. Then that would improve the readability and justify the use of a mixed-methods approach to assess the challenges in preparedness for public health emergencies.	Thanks for the comment. Since the KII findings were analyzed and presented with deductive qualitative approach with Us CDC framework, authors feel that keeping those separately would enhance the comparability with other settings. However considering this comment, better integration of the findings was done	Methods and Results sections

	in the revised manuscript.	
Discussion<br>A more in-depth analysis of the global literature would improve this paper. For example, statements such as “many of the other policy-related challenges identified in this study are aligned with those have been reported globally” (lines 287–288) are too general and should be explored in more detail. This would help a reader understand how Sri Lanka's challenges are similar to other countries or if they are unique.	Thanks for the comment. More in-depth analysis was done in the discussion section, considering this comment and the comments of editor and other reviewer.	Discussion section
Grammar, language formatting and other errors<br>Inconsistent date:<br>There is a discrepancy in the dates of the when the KIIs were conducted “from March to June 2025” (lines 43–44),but the Methods section (line 144) states they were conducted “between 1st April and 31st May<br>2025	Thanks for the comment. The duration has been corrected as April to May 2025	Methods- 5th paragraph
Table numbering error:<br>Tables are numbered out of order: Table 1 (KII breakdown), then Table 2 (stakeholder grid), then Table 4 (policy document gaps), then Table 3 (KII challenges).	Thanks for the comment. Table numbering has been corrected in the revised manuscript	Table numbers
• Line 29: “facilitate” should be “facilitates” (subject-verb agreement with “coordination”).<br>• Line 68: “coordinate” should be “coordinated.”<br>• Line 90: Missing period after the closing quotation mark.<br>• Line 94: Missing period after “(11)”.<br>• Line 311: “fragmentism” is not a standard English word; use “fragmentation.” Also “anad”<br>should be “and.”<br>• Line 377: “preapredness” should be “preparedness.”<br>• Line 333: awkward phrasing “it was elicited was that” – remove one “was.”<br>• Lines 43–44: Missing period after “June 2025” in the abstract.<br>• Table 3, People domain: “No common training for a are found” contains a typographical error<br>(“for a” should likely be “fora”).<br>Annex 2 lists the Central Province district “Malate”	Thanks for pointing out these. All corrections are done in the revised manuscript	Throughout the manuscript

– this should be "Matale."<br>• "Hambanthotha" in Annex 2 should be "Hambantota."<br>• "Gamapaha" in Annex 2 should be "Gampaha."		

Reviewer #2:

**Comment**	**Action/s taken**	**Section in revised manuscript**
Thank you for the invitation to review this manuscript which presents a multi-phase project focused on multisectoral preparedness and response for public health emergencies in Sri Lanka. The article reports an important body of work that was carried out nationally, uses a range of methods across four study phases (risk assessment, stakeholder mapping, desk review and key informant interviews), and was guided by the US CDC model on public health linkages as a conceptual framework. The integrated study design and use of a conceptual framework are particular strengths of the paper. The rationale for the project is well explained and convincing. There are important issues of coherence and alignment in the methods and presentation of the results that require attention and, to strengthen the contribution of the paper, I recommend expanding on the integrated analysis that looks transversally across the study phases.	Thanks for the encouraging and insightful comments. Necessary modifications have been done in the revised manuscript as elaborated under each of the below comments.	Throughout the manuscript
Abstract:<br>The initial methods sentence identifies the different data sources, with subsequent sentences focused on each data source/method. There is some inconsistency in describing what was done and for which purpose for each phase (sometimes we are told about the aim and sometimes about the method, for example). I recommend naming the data sources in the first sentence and then giving both 'what' and 'why' information in each following sentence. Also, 'who' participated in KII interviews should be clarified here.	Thanks for the comment. The abstract has been revised as per these comments of the reviewer in the revised manuscript.	Abstract

Data availability statement: This statement does not appear accurate given the nature of the study data and inclusion of the KII interviews in particular. This data may not be able to be made available due to confidentiality commitments, but the statement as written does not reflect this.	Thanks for the comment. The data availability statement revised to reflect that data on KII and personal opinions will be made available without details leading to identification of the responder.	Data availability statement
Introduction		
1) Definition of PHE: The notion of "challenging" the health system seems insufficient to convey the gravity of a PHE. Nelson et al are cited here, and they specifically focus on those situations where there is a risk that routine capacities will be overwhelmed.	Thanks for the comment. "With routine capacities been overwhelmed" phrase was added in the revised manuscript.	Introduction- 1st paragraph
2) The notion of health system "building blocks" is mentioned twice in the first paragraph. What do these entail? Best to list categories or give key examples.	Thanks for the comment. The six building blocks have been listed in the 1st paragraph.	Introduction- 1st paragraph
3) Likewise, "multisectoral action" is discussed at length in the introduction and is a central concept for the paper, so would benefit from a clear definition early on, including identifying the range of sectors that are the focus of the analysis developed here.	Thanks for the comment. Multisectoral action has been defined in the revised manuscript.	Introduction – 3rd paragraph
Methods:		
Risk assessment: it is not clear how many documents were included and how input from experts was collected and integrated. No methodological references are given to support the approach taken and we do not get a clear sense of how risks are actually going to be assessed.	Thanks for the comment. This was done with actual frequency/ potential likelihood and impact. Necessary revisions have been done in the revised manuscript.	Methods- 2nd paragraph

Stakeholder mapping, Line 131: Who are the "relevant officers" referred to here?	Thanks for the comment. They were "technical experts in the institutions who are related to multisectoral actions". This was added to the revised manuscript.	Methods- 3rd paragraph
Desk review:<br>There is a major issue of consistency in that the description of the desk review's objective and steps do not match what is stated in the abstract ("The desk review aimed to identify the deficiencies of the existing major policy documents for multisectoral action"), nor the material included in the results section.	Thanks for the comment. The desk review included the literature search and review of reports available at institutions. These have been shown in Table 3. Manuscript has been revised to make these clearer.	Methods section
It is helpful to have the annex with the search strategy in PubMed, yielding 2245 documents for review. How many documents were screened through searches in Google Scholar (and using which search strings?) or gray literature sources recommended by experts? Additional details about the search and screening processes will help the reader to have a better sense of the scope of the endeavour. This information could be included in the annex to fill out the description of the review, including a flowchart.	Thanks for the comment. More details on the Google Scholar search was added in the revised manuscript.	Methods section
Key informant interviews: The authors state the interviews were "conducted… following recommended methodology." However, the references given are for two papers on focus groups methods, rather than key informant interviews, and several papers on trustworthiness in qualitative research. There should be a clearer description of what qualitative research approach/methodology is being used and alignment of the references with the methods employed. In terms of steps to ensure trustworthiness, it would be better to show this in the description of the methods rather than claim it.	Thanks for the comment. Additional details have been added in the revised manuscript on KIIs.	Methods section- 5th paragraph

Some reordering of material would help to follow the logical flow of such a project: First, we should hear about the rationale (sampling strategy) and the process (recruitment steps) for seeking out participants, before the actual characteristics of those selected. The nature of the purposive sampling should be explained so the reader understands, for example, why the participant category from PDHS and RDHS (28) is vastly more represented than other participant categories. How long were the interviews (range or average)?		
Data analysis: This description is insufficient for the reader to understand how analysis was undertaken within the different components of the project and then the analysis brought together for the convergent analysis across components.	Thanks for the comment. The analysis section has been more elaborated in the revised manuscript.	Methods section
Results:		
The names of sub-headings are not always inclusive of all material included under them (e.g. policy analysis is included under sub-heading "Actual and potential PHEs of Sri Lanka and Stakeholder grid").	Thanks for the comment. Sub-headings were revised	Results section
Risk assessment: The list of PHEs in the past 10 years could be more informative if it also included a sense of frequency and degree of impact. It is also not clear here or in the methods how potential event risks were determined. More broadly, a list of past and potential PHE triggers seems like a limited form of risk assessment. My impression is that this aspect of the project is relatively thin in terms of both explanation of process and what it yields in terms of knowledge. Providing more detail on methods and layering in additional results would make the contribution of this phase of the study more evident.	Thanks for the comment. As mentioned the risk assessment was based on the data available at Disaster Preparedness and Response Division, Provincial Directorates and Regional Directorates. The technical experts in these units were consulted and asked about the actual frequency/potential frequency and impact of the event. These are added in	Methods section-2nd paragraph

	the revised manuscript.	
Stakeholder mapping: There are many different actors included in the stakeholder mapping exercise but it is striking that all but two of them are categorized as high/high. How was it decided which entities to include in the mapping (i.e. how wide to cast the net for the mapping exercise)? Non-governmental organizations (and likely also universities/academics) are diverse in terms of focus and orientation so it could be challenging to include all such actors in a single category.	The data obtained from the experts in the risk assessment was used in this process. The revised manuscript gives a clear description of this.	Methods section-2nd paragraph
Desk review: the results here – eight national policy documents – do not match with what is described in the methods section, which announced an intent for the desk review to "collate evidence for facilitators and barriers for multisectoral action and its governance." The search does not appear to be designed to surface policy documents in particular (though they are mentioned as possible "additional grey literature) and the process of categorizing and analyzing policies is not described in the methods.	The desk review included a literature review and review of reports. It was by these that the eight policy documents were identified. The manuscript was revised to make this clearer.	Methods section
Key informant interviews: Line 238: in what way is this "building on the limitations identified in the desk review"? 51 key informant interviews are likely to generate a wealth of information related to the topic, so it is surprising to only have lists of challenges across the four domains. Do the authors plan to publish further results from this component of the project separately?	KIIs were done to elicit the challenges associated with the existing policy deficiencies. Another domain covered in the KIIs was developing the priority-actions based on the suggestions to address these challenges, which will be dealt in a separate manuscript. Under the deductive approach, the challenges were asked in the semi-structured questionnaire under	Methods section

	the 4 domains of the US CDC model. The manuscript was revised to highlight these aspects.	
Collated analysis<br>L254: This sentence refers to a literature review and a desk review. In the methods section, the desk review IS a literature review. Also, it would be better to describe this step in terms of a convergent analysis across phases of the project (as was announced by the authors at the end of the methods), rather than collating evidence.	Thanks for the comment. The desk review included a literature review and review of reports. The manuscript was revised to make this clearer	Methods and Results sections
The final paragraph of the results which provides a more integrated analysis provides the richest portrait of the situation. It would be very interesting to have the authors expand on this analysis by fleshing out the insights generated by looking across the different components of their project and in relation to these main challenges.	Thanks for the comment. Linkage has been done with the final paragraph and the discussion section especially with the newly added Table 5.	Discussion section
Discussion		
This section helpfully puts the study findings in context and offers a series of recommendations building up from the results. I suggest adding a table or figure that gathers up all the main recommendations.	Thanks for the comment. Table 5 was added gathering up main recommendations	Table 5
This statement is not clear: “Furthermore a consistent prioritization technique was used that helped standardize inputs more representative.” The technique is not mentioned in the methods and it is not clear what it entails, nor what purpose it served.	This was removed as it is more related to the companion manuscript which is on setting priority actions.	Limitations
Do the authors have reflections or recommendations regarded the methods they chose and how they were implemented? For example, reflections on the scope of the stakeholder mapping or including KIIs from other groups? Are there lessons that can be drawn for others who may wish to implement a similar process in another country (e.g. steps that could be added, obstacles to avoid)?	Thanks for the comment. The second paragraph of the revised manuscript now include reflections of the methodology.	Discussion-second paragraph

Overall:		
The article is well written and has a clear structure. There are occasional grammatical errors and missing words in the text that should be attended to with a close copy edit.	Thanks for the comment. Language corrections were done while considering the comments of the other reviewer as well	Throughout the manuscript
