# Supplementary material for: Challenges in strengthening multi-sectoral action for optimum preparedness and response for public health emergencies in Sri Lanka
Source: PLOS Glob Public Health. 2026 Jul 7;6(7):e0005964. doi: 10.1371/journal.pgph.0005964 (PMC13340774; doi:10.1371/journal.pgph.0005964)
Supplement: Attachment — Submitted filename: Respons to Reviewers - 2026.04.25.docx [file pgph.0005964.s004.docx]

**Response to the comments of reviewers**

**Editor**

| **Comment** | **Actions taken** | **Section in revised manuscript** |
| --- | --- | --- |
| Thank you for conducting this timely and impactful research - which will be highly relevant given the current context of Sri Lanka trying to strengthen disaster response and recovery - also will have strong relevance for the future as climate crisis induced PHEs will increase. I agree with the reviewers that clarity in methods and overall coherence could strengthen this paper. Please pay close attention to Reviewer 2 comments on strengthening the methods and coherence of the paper. This is an important paper for policy - and strengthening the methods and coherence will improve the impact the paper can have and academic credibility in terms of research rigour. | Thanks for the comment. All comments of the both reviewers have been addressed as shown below. Close attention was paid to the comments of reviewer 2 on strengthening the methods and the coherence of the paper. | Throughout the manuscript |
| Additionally, if possible, I also suggest strengthening the discussion section by bringing in the recent cyclone Ditwah into the discussion section. Although, this is a natural disaster at first, as we know natural disasters invariable have an impact on public health (and fall within the definition provided). Including the recent natural disaster in the discussion section and where necessary in conclusion/recommendation will increase the relevance of this paper and improve the uptake in national, regional and global settings. Furthermore, as NGO coordination and other challenges are mentioned and given the role NGOs play in disaster responses - would be good to discuss NGO role and pathways for strengthening that role. | Thanks for the comment. The discussion was made enriched with references to the Ditwah as well as on NGO role. | Discussion section |

**Reviewer #1:** 

| **Comment** | **Action/s taken** | **Section in revised manuscript** |
| --- | --- | --- |
| I commend the authors for doing a deep-dive in addressing a relevant and timely topic. Overall, I liked the mixed method approach involving risk assessment, stakeholder mapping, desk review and hey informant interviews which I thought was quite comprehensive | Thanks for the encouraging comment | NA |
| The KII interviews:  The authors were also from the Ministry of Health Sri Lanka which creates a bias that is not addressed in the manuscript.  It would be better if more details about the interviews (duration, language) were explicitly mentioned. Also mention whether the transcribing was done by two authors independently which would remove biases. The questionnaire should be made available. It would strengthen the study if direct quotes from the interviewees were also included. | Thanks for the comment. The necessary details have been added. The questionnaire has been made available as Annex 4. | Methods section |
| Desk review:  I recommend that the authors integrate the desk review findings and KII interview findings instead of presenting them sequentially. Then that would improve the readability and justify the use of a mixed-methods approach to assess the challenges in preparedness for public health emergencies. | Thanks for the comment. Since the KII findings were analyzed and presented with deductive qualitative approach with Us CDC framework, authors feel that keeping those separately would enhance the comparability with other settings. However considering this comment, better integration of the findings was done in the revised manuscript. | Methods and Results sections |
| Discussion A more in-depth analysis of the global literature would improve this paper. For example, statements such as “many of the other policy-related challenges identified in this study are aligned with those have been reported globally” (lines 287–288) are too general and should be explored in more detail. This would help a reader understand how Sri Lanka's challenges are similar to other countries or if they are unique. | Thanks for the comment. More in-depth analysis was done in the discussion section, considering this comment and the comments of editor and other reviewer. | Discussion section |
| Grammar, language formatting and other errors Inconsistent date: There is a discrepancy in the dates of the when the KIIs were conducted “from March to June 2025” (lines 43–44),but the Methods section (line 144) states they were conducted “between 1st April and 31st May 2025 | Thanks for the comment. The duration has been corrected as April to May 2025 | Methods- 5^th^ paragraph |
| Table numbering error: Tables are numbered out of order: Table 1 (KII breakdown), then Table 2 (stakeholder grid), then Table 4 (policy document gaps), then Table 3 (KII challenges). | Thanks for the comment. Table numbering has been corrected in the revised manuscript | Table numbers |
| • Line 29: “facilitate” should be “facilitates” (subject-verb agreement with “coordination”). • Line 68: “coordinate” should be “coordinated.” • Line 90: Missing period after the closing quotation mark. • Line 94: Missing period after “(11)”. • Line 311: “fragmentism” is not a standard English word; use “fragmentation.” Also “anad” should be “and.” • Line 377: “preapredness” should be “preparedness.” • Line 333: awkward phrasing “it was elicited was that” – remove one “was.” • Lines 43–44: Missing period after “June 2025” in the abstract. • Table 3, People domain: “No common training for a are found” contains a typographical error (“for a” should likely be “fora”). Annex 2 lists the Central Province district “Malate” – this should be “Matale.” • “Hambanthotha” in Annex 2 should be “Hambantota.” • “Gamapaha” in Annex 2 should be “Gampaha.” | Thanks for pointing out these. All corrections are done in the revised manuscript | Throughout the manuscript |

**Reviewer #2:**

| **Comment** | **Action/s taken** | **Section in revised manuscript** |
| --- | --- | --- |
| Thank you for the invitation to review this manuscript which presents a multi-phase project focused on multisectoral preparedness and response for public health emergencies in Sri Lanka. The article reports an important body of work that was carried out nationally, uses a range of methods across four study phases (risk assessment, stakeholder mapping, desk review and key informant interviews), and was guided by the US CDC model on public health linkages as a conceptual framework. The integrated study design and use of a conceptual framework are particular strengths of the paper. The rationale for the project is well explained and convincing. There are important issues of coherence and alignment in the methods and presentation of the results that require attention and, to strengthen the contribution of the paper, I recommend expanding on the integrated analysis that looks transversally across the study phases. | Thanks for the encouraging and insightful comments. Necessary modifications have been done in the revised manuscript as elaborated under each of the below comments. | Throughout the manuscript |
| Abstract: The initial methods sentence identifies the different data sources, with subsequent sentences focused on each data source/method. There is some inconsistency in describing what was done and for which purpose for each phase (sometimes we are told about the aim and sometimes about the method, for example). I recommend naming the data sources in the first sentence and then giving both ‘what’ and ‘why’ information in each following sentence. Also, ‘who’ participated in KII interviews should be clarified here. | Thanks for the comment. The abstract has been revised as per these comments of the reviewer in the revised manuscript. | Abstract |
| Data availability statement: This statement does not appear accurate given the nature of the study data and inclusion of the KII interviews in particular. This data may not be able to be made available due to confidentiality commitments, but the statement as written does not reflect this. | Thanks for the comment. The data availability statement revised to reflect that data on KII and personal opinions will be made available without details leading to identification of the responder. | Data availability statement |
| Introduction |  |  |
| 1) Definition of PHE: The notion of “challenging” the health system seems insufficient to convey the gravity of a PHE. Nelson et al are cited here, and they specifically focus on those situations where there is a risk that routine capacities will be overwhelmed. | Thanks for the comment. “With routine capacities been overwhelmed” phrase was added in the revised manuscript. | Introduction- 1^st^ paragraph |
| 2) The notion of health system “building blocks” is mentioned twice in the first paragraph. What do these entail? Best to list categories or give key examples. | Thanks for the comment. The six building blocks have been listed in the 1^st^ paragraph. | Introduction- 1st paragraph |
| 3) Likewise, “multisectoral action” is discussed at length in the introduction and is a central concept for the paper, so would benefit from a clear definition early on, including identifying the range of sectors that are the focus of the analysis developed here. | Thanks for the comment. Multisectoral action has been defined in the revised manuscript. | Introduction – 3^rd^ paragraph |
| Methods: |  |  |
| Risk assessment: it is not clear how many documents were included and how input from experts was collected and integrated. No methodological references are given to support the approach taken and we do not get a clear sense of how risks are actually going to be assessed. | Thanks for the comment. This was done with actual frequency/ potential likelihood and impact. Necessary revisions have been done in the revised manuscript. | Methods- 2^nd^ paragraph |
| Stakeholder mapping, Line 131: Who are the “relevant officers” referred to here? | Thanks for the comment. They were “technical experts in the institutions who are related to multisectoral actions”. This was added to the revised manuscript. | Methods- 3^rd^ paragraph |
| Desk review: There is a major issue of consistency in that the description of the desk review’s objective and steps do not match what is stated in the abstract (“The desk review aimed to identify the deficiencies of the existing major policy documents for multisectoral action”), nor the material included in the results section. | Thanks for the comment. The desk review included the literature search and review of reports available at institutions. These have been shown in Table 3. Manuscript has been revised to make these clearer. | Methods section |
| It is helpful to have the annex with the search strategy in PubMed, yielding 2245 documents for review. How many documents were screened through searches in Google Scholar (and using which search strings?) or gray literature sources recommended by experts? Additional details about the search and screening processes will help the reader to have a better sense of the scope of the endeavour. This information could be included in the annex to fill out the description of the review, including a flowchart. | Thanks for the comment. More details on the Google Scholar search was added in the revised manuscript. | Methods section |
| Key informant interviews: The authors state the interviews were “conducted… following recommended methodology.” However, the references given are for two papers on focus groups methods, rather than key informant interviews, and several papers on trustworthiness in qualitative research. There should be a clearer description of what qualitative research approach/methodology is being used and alignment of the references with the methods employed. In terms of steps to ensure trustworthiness, it would be better to show this in the description of the methods rather than claim it. Some reordering of material would help to follow the logical flow of such a project: First, we should hear about the rationale (sampling strategy) and the process (recruitment steps) for seeking out participants, before the actual characteristics of those selected. The nature of the purposive sampling should be explained so the reader understands, for example, why the participant category from PDHS and RDHS (28) is vastly more represented than other participant categories. How long were the interviews (range or average)? | Thanks for the comment. Additional details have been added in the revised manuscript on KIIs. | Methods section- 5^th^ paragraph |
| Data analysis: This description is insufficient for the reader to understand how analysis was undertaken within the different components of the project and then the analysis brought together for the convergent analysis across components. | Thanks for the comment. The analysis section has been more elaborated in the revised manuscript. | Methods section |
| Results: |  |  |
| The names of sub-headings are not always inclusive of all material included under them (e.g. policy analysis is included under sub-heading “Actual and potential PHEs of Sri Lanka and Stakeholder grid”). | Thanks for the comment. Sub-headings were revised | Results section |
| Risk assessment: The list of PHEs in the past 10 years could be more informative if it also included a sense of frequency and degree of impact. It is also not clear here or in the methods how potential event risks were determined. More broadly, a list of past and potential PHE triggers seems like a limited form of risk assessment. My impression is that this aspect of the project is relatively thin in terms of both explanation of process and what it yields in terms of knowledge. Providing more detail on methods and layering in additional results would make the contribution of this phase of the study more evident. | Thanks for the comment. As mentioned the risk assessment was based on the data available at Disaster Preparedness and Response Division, Provincial Directorates and Regional Directorates. The technical experts in these units were consulted and asked about the actual frequency/potential frequency and impact of the event. These are added in the revised manuscript. | Methods section- 2nd paragraph |
| Stakeholder mapping: There are many different actors included in the stakeholder mapping exercise but it is striking that all but two of them are categorized as high/high. How was it decided which entities to include in the mapping (i.e. how wide to cast the net for the mapping exercise)? Non-governmental organizations (and likely also universities/academics) are diverse in terms of focus and orientation so it could be challenging to include all such actors in a single category. | The data obtained from the experts in the risk assessment was used in this process. The revised manuscript gives a clear description of this. | Methods section- 2^nd^ paragraph |
| Desk review: the results here – eight national policy documents – do not match with what is described in the methods section, which announced an intent for the desk review to “collate evidence for facilitators and barriers for multisectoral action and its governance.” The search does not appear to be designed to surface policy documents in particular (though they are mentioned as possible “additional grey literature) and the process of categorizing and analyzing policies is not described in the methods. | The desk review included a literature review and review of reports. It was by these that the eight policy documents were identified. The manuscript was revised to make this clearer. | Methods section |
| Key informant interviews: Line 238: in what way is this “building on the limitations identified in the desk review”? 51 key informant interviews are likely to generate a wealth of information related to the topic, so it is surprising to only have lists of challenges across the four domains. Do the authors plan to publish further results from this component of the project separately? | KIIs were done to elicit the challenges associated with the existing policy deficiencies. Another domain covered in the KIIs was developing the priority-actions based on the suggestions to address these challenges, which will be dealt in a separate manuscript. Under the deductive approach, the challenges were asked in the semi-structured questionnaire under the 4 domains of the US CDC model. The manuscript was revised to highlight these aspects. | Methods section |
| Collated analysis L254: This sentence refers to a literature review and a desk review. In the methods section, the desk review IS a literature review. Also, it would be better to describe this step in terms of a convergent analysis across phases of the project (as was announced by the authors at the end of the methods), rather than collating evidence. | Thanks for the comment. The desk review included a literature review and review of reports. The manuscript was revised to make this clearer | Methods and Results sections |
| The final paragraph of the results which provides a more integrated analysis provides the richest portrait of the situation. It would be very interesting to have the authors expand on this analysis by fleshing out the insights generated by looking across the different components of their project and in relation to these main challenges. | Thanks for the comment. Linkage has been done with the final paragraph and the discussion section especially with the newly added Table 5. | Discussion section |
| Discussion |  |  |
| This section helpfully puts the study findings in context and offers a series of recommendations building up from the results. I suggest adding a table or figure that gathers up all the main recommendations. | Thanks for the comment. Table 5 was added gathering up main recommendations | Table 5 |
| This statement is not clear: “Furthermore a consistent prioritization technique was used that helped standardize inputs more representative.” The technique is not mentioned in the methods and it is not clear what it entails, nor what purpose it served. | This was removed as it is more related to the companion manuscript which is on setting priority actions. | Limitations |
| Do the authors have reflections or recommendations regarded the methods they chose and how they were implemented? For example, reflections on the scope of the stakeholder mapping or including KIIs from other groups? Are there lessons that can be drawn for others who may wish to implement a similar process in another country (e.g. steps that could be added, obstacles to avoid)? | Thanks for the comment. The second paragraph of the revised manuscript now include reflections of the methodology. | Discussion- second paragraph |
| Overall: |  |  |
| The article is well written and has a clear structure. There are occasional grammatical errors and missing words in the text that should be attended to with a close copy edit. | Thanks for the comment. Language corrections were done while considering the comments of the other reviewer as well | Throughout the manuscript |
