# Supplementary material for: Challenges in strengthening multi-sectoral action for optimum preparedness and response for public health emergencies in Sri Lanka
Source: PLOS Glob Public Health. 2026 Jul 7;6(7):e0005964. doi: 10.1371/journal.pgph.0005964 (PMC13340774; doi:10.1371/journal.pgph.0005964)
Supplement: Attachment — Submitted filename: Respons to reviewers -28.05.2026.docx [file pgph.0005964.s006.docx]

**Response to the comments of reviewers for the first revision**

**Editor**

| Comment | Actions taken | Section in revised manuscript |
| --- | --- | --- |
| Thanks for taking the time to comprehensively revise the paper. The methods and approach is markedly clearer. However, there are some gaps in clarity and consistency of presentation of the paper. To make it easier for the authorship team, I have made comments in the tracked changes version of the paper (Revision 1) with suggested edits and relevant reframing. Where relevant I have suggested additional citations (two places). Since this paper will be read by a global audience, I have made edits to ensure a generalist, global audience can also understand the results presented. I believe these edits are relatively easy to make. If you have questions as you revise, please reach out to me - I will be happy to support with clarifications. | Many thanks for the encouragement. Authors are extremely grateful for the handling editor for the comprehensive review and for the suggestions provided for almost of the comments.  All the suggestions (including 3 references) have been incorporated in this second-revised version | Throughout the manuscript |
