# Supplementary material for: Challenges in strengthening multi-sectoral action for optimum preparedness and response for public health emergencies in Sri Lanka
Source: PLOS Glob Public Health. 2026 Jul 7;6(7):e0005964. doi: 10.1371/journal.pgph.0005964 (PMC13340774; doi:10.1371/journal.pgph.0005964)
Supplement: Peer review history

# Peer review: Decision Letter 0

**DOI:** 10.1371/journal.pgph.0005964.r001

**Authors:** Shashika Bandara

15 Mar 2026

PGPH-D-26-00295

Challenges in strengthening multi-sectoral action for optimum preparedness and response for public health emergencies in Sri Lanka

PLOS Global Public Health

Dear Dr. Arnold,

Thank you for submitting your manuscript to PLOS Global Public Health. After careful consideration, we feel that it has merit but does not fully meet PLOS Global Public Health’s publication criteria as it currently stands. Therefore, we invite you to submit a revised version of the manuscript that addresses the points raised during the review process.

Please submit your revised manuscript by Apr 29 2026 11:59PM. If you will need more time than this to complete your revisions, please reply to this message or contact the journal office at globalpubhealth@plos.org. When you're ready to submit your revision, log on to https://www.editorialmanager.com/pgph/ and select the 'Submissions Needing Revision' folder to locate your manuscript file.

Please include the following items when submitting your revised manuscript:

- A letter that responds to each point raised by the editor and reviewer(s). You should upload this letter as a separate file labeled 'Response to Reviewers'.
- A marked-up copy of your manuscript that highlights changes made to the original version. You should upload this as a separate file labeled 'Revised Manuscript with Track Changes'.
- An unmarked version of your revised paper without tracked changes. You should upload this as a separate file labeled 'Manuscript'.

Guidelines for resubmitting your figure files are available below the reviewer comments at the end of this letter.

We look forward to receiving your revised manuscript.

Kind regards,

Shashika Bandara

Academic Editor

PLOS Global Public Health

Journal Requirements:

If the reviewer comments include a recommendation to cite specific previously published works, please review and evaluate these publications to determine whether they are relevant and should be cited. There is no requirement to cite these works unless the editor has indicated otherwise.

Additional Editor Comments (if provided):

Dear Authors,

Thank you for conducting this timely and impactful research - which will be highly relevant given the current context of Sri Lanka trying to strengthen disaster response and recovery - also will have strong relevance for the future as climate crisis induced PHEs will increase. I agree with the reviewers that clarity in methods and overall coherence could strengthen this paper. Please pay close attention to Reviewer 2 comments on strengthening the methods and coherence of the paper. This is an important paper for policy - and strengthening the methods and coherence will improve the impact the paper can have and academic credibility in terms of research rigour.

Additionally, if possible, I also suggest strengthening the discussion section by bringing in the recent cyclone Ditwah into the discussion section. Although, this is a natural disaster at first, as we know natural disasters invariable have an impact on public health (and fall within the definition provided). Including the recent natural disaster in the discussion section and where necessary in conclusion/recommendation will increase the relevance of this paper and improve the uptake in national, regional and global settings. Furthermore, as NGO coordination and other challenges are mentioned and given the role NGOs play in disaster responses - would be good to discuss NGO role and pathways for strengthening that role.

Looking forward to receiving the revised paper.

Shashika

[Note: HTML markup is below. Please do not edit.]

Reviewers' comments:

Reviewer's Responses to Questions

**Comments to the Author**

1. Does this manuscript meet PLOS Global Public Health’s publication criteria? Is the manuscript technically sound, and do the data support the conclusions? The manuscript must describe methodologically and ethically rigorous research with conclusions that are appropriately drawn based on the data presented.

Reviewer #1: Partly

Reviewer #2: Partly

**********

2. Has the statistical analysis been performed appropriately and rigorously?

Reviewer #1: N/A

Reviewer #2: N/A

**********

3. Have the authors made all data underlying the findings in their manuscript fully available (please refer to the Data Availability Statement at the start of the manuscript PDF file)?

The PLOS Data policy requires authors to make all data underlying the findings described in their manuscript fully available without restriction, with rare exception. The data should be provided as part of the manuscript or its supporting information, or deposited to a public repository. For example, in addition to summary statistics, the data points behind means, medians and variance measures should be available. If there are restrictions on publicly sharing data—e.g. participant privacy or use of data from a third party—those must be specified.

Reviewer #1: No

Reviewer #2: No

**********

4. Is the manuscript presented in an intelligible fashion and written in standard English?

PLOS Global Public Health does not copyedit accepted manuscripts, so the language in submitted articles must be clear, correct, and unambiguous. Any typographical or grammatical errors should be corrected at revision, so please note any specific errors here.

Reviewer #1: Yes

Reviewer #2: Yes

**********

5. Review Comments to the Author

Please use the space provided to explain your answers to the questions above. You may also include additional comments for the author, including concerns about dual publication, research ethics, or publication ethics. (Please upload your review as an attachment if it exceeds 20,000 characters)

Reviewer #1: I commend the authors for doing a deep-dive in addressing a relevant and timely topic. Overall, I liked the mixed method approach involving risk assessment, stakeholder mapping, desk review and hey informant interviews which I thought was quite comprehensive.

I have a few suggestions and comments to improve this manuscript prior to publication.

The KII interviews:

The authors were also from the Ministry of Health Sri Lanka which creates a bias that is not addressed in the manuscript.

It would be better if more details about the interviews (duration, language) were explicitly mentioned. Also mention whether the transcribing was done by two authors independently which would remove biases. The questionnaire should be made available. It would strengthen the study if direct quotes from the interviewees were also included.

Desk review

I recommend that the authors integrate the desk review findings and KII interview findings instead of presenting them sequentially. Then that would improve the readability and justify the use of a mixed-methods approach to assess the challenges in preparedness for public health emergencies.

Discussion

A more in-depth analysis of the global literature would improve this paper. For example, statements such as “many of the other policy-related challenges identified in this study are aligned with those have been reported globally” (lines 287–288) are too general and should be explored in more detail. This would help a reader understand how Sri Lanka's challenges are similar to other countries or if they are unique.

Grammar, language formatting and other errors

Inconsistent date:

There is a discrepancy in the dates of the when the KIIs were conducted “from March to June 2025” (lines 43–44),but the Methods section (line 144) states they were conducted “between 1st April and 31st May

2025.”

Table numbering error:

Tables are numbered out of order: Table 1 (KII breakdown), then Table 2 (stakeholder grid), then

Table 4 (policy document gaps), then Table 3 (KII challenges).

• Line 29: “facilitate” should be “facilitates” (subject-verb agreement with “coordination”).

• Line 68: “coordinate” should be “coordinated.”

• Line 90: Missing period after the closing quotation mark.

• Line 94: Missing period after “(11)”.

• Line 311: “fragmentism” is not a standard English word; use “fragmentation.” Also “anad”

should be “and.”

• Line 377: “preapredness” should be “preparedness.”

• Line 333: awkward phrasing “it was elicited was that” – remove one “was.”

• Lines 43–44: Missing period after “June 2025” in the abstract.

• Table 3, People domain: “No common training for a are found” contains a typographical error

(“for a” should likely be “fora”).

Annex 2 lists the Central Province district “Malate” – this should be “Matale.”

• “Hambanthotha” in Annex 2 should be “Hambantota.”

• “Gamapaha” in Annex 2 should be “Gampaha.”

Reviewer #2: Thank you for the invitation to review this manuscript which presents a multi-phase project focused on multisectoral preparedness and response for public health emergencies in Sri Lanka. The article reports an important body of work that was carried out nationally, uses a range of methods across four study phases (risk assessment, stakeholder mapping, desk review and key informant interviews), and was guided by the US CDC model on public health linkages as a conceptual framework. The integrated study design and use of a conceptual framework are particular strengths of the paper. The rationale for the project is well explained and convincing. There are important issues of coherence and alignment in the methods and presentation of the results that require attention and, to strengthen the contribution of the paper, I recommend expanding on the integrated analysis that looks transversally across the study phases. Below, I make recommendations that I believe could help the authors in revising their paper.

Abstract:

The initial methods sentence identifies the different data sources, with subsequent sentences focused on each data source/method. There is some inconsistency in describing what was done and for which purpose for each phase (sometimes we are told about the aim and sometimes about the method, for example). I recommend naming the data sources in the first sentence and then giving both ‘what’ and ‘why’ information in each following sentence. Also, ‘who’ participated in KII interviews should be clarified here.

Data availability statement: This statement does not appear accurate given the nature of the study data and inclusion of the KII interviews in particular. This data may not be able to be made available due to confidentiality commitments, but the statement as written does not reflect this.

The introduction lays out important background information to the study. Further attention to clarifying key concepts would help the reader:

1) Definition of PHE: The notion of “challenging” the health system seems insufficient to convey the gravity of a PHE. Nelson et al are cited here, and they specifically focus on those situations where there is a risk that routine capacities will be overwhelmed.

2) The notion of health system “building blocks” is mentioned twice in the first paragraph. What do these entail? Best to list categories or give key examples.

3) Likewise, “multisectoral action” is discussed at length in the introduction and is a central concept for the paper, so would benefit from a clear definition early on, including identifying the range of sectors that are the focus of the analysis developed here.

Methods:

Risk assessment: it is not clear how many documents were included and how input from experts was collected and integrated. No methodological references are given to support the approach taken and we do not get a clear sense of how risks are actually going to be assessed.

Stakeholder mapping, Line 131: Who are the “relevant officers” referred to here?

Desk review:

There is a major issue of consistency in that the description of the desk review’s objective and steps do not match what is stated in the abstract (“The desk review aimed to identify the deficiencies of the existing major policy documents for multisectoral action”), nor the material included in the results section.

It is helpful to have the annex with the search strategy in PubMed, yielding 2245 documents for review. How many documents were screened through searches in Google Scholar (and using which search strings?) or gray literature sources recommended by experts? Additional details about the search and screening processes will help the reader to have a better sense of the scope of the endeavour. This information could be included in the annex to fill out the description of the review, including a flowchart.

Key informant interviews: The authors state the interviews were “conducted… following recommended methodology.” However, the references given are for two papers on focus groups methods, rather than key informant interviews, and several papers on trustworthiness in qualitative research. There should be a clearer description of what qualitative research approach/methodology is being used and alignment of the references with the methods employed. In terms of steps to ensure trustworthiness, it would be better to show this in the description of the methods rather than claim it.

Some reordering of material would help to follow the logical flow of such a project: First, we should hear about the rationale (sampling strategy) and the process (recruitment steps) for seeking out participants, before the actual characteristics of those selected. The nature of the purposive sampling should be explained so the reader understands, for example, why the participant category from PDHS and RDHS (28) is vastly more represented than other participant categories. How long were the interviews (range or average)?

Data analysis: This description is insufficient for the reader to understand how analysis was undertaken within the different components of the project and then the analysis brought together for the convergent analysis across components.

Results:

The names of sub-headings are not always inclusive of all material included under them (e.g. policy analysis is included under sub-heading “Actual and potential PHEs of Sri Lanka and Stakeholder grid”).

Risk assessment: The list of PHEs in the past 10 years could be more informative if it also included a sense of frequency and degree of impact. It is also not clear here or in the methods how potential event risks were determined. More broadly, a list of past and potential PHE triggers seems like a limited form of risk assessment. My impression is that this aspect of the project is relatively thin in terms of both explanation of process and what it yields in terms of knowledge. Providing more detail on methods and layering in additional results would make the contribution of this phase of the study more evident.

Stakeholder mapping: There are many different actors included in the stakeholder mapping exercise but it is striking that all but two of them are categorized as high/high. How was it decided which entities to include in the mapping (i.e. how wide to cast the net for the mapping exercise)? Non-governmental organizations (and likely also universities/academics) are diverse in terms of focus and orientation so it could be challenging to include all such actors in a single category.

Desk review: the results here – eight national policy documents – do not match with what is described in the methods section, which announced an intent for the desk review to “collate evidence for facilitators and barriers for multisectoral action and its governance.” The search does not appear to be designed to surface policy documents in particular (though they are mentioned as possible “additional grey literature) and the process of categorizing and analyzing policies is not described in the methods.

Key informant interviews: Line 238: in what way is this “building on the limitations identified in the desk review”? 51 key informant interviews are likely to generate a wealth of information related to the topic, so it is surprising to only have lists of challenges across the four domains. Do the authors plan to publish further results from this component of the project separately?

Collated analysis

L254: This sentence refers to a literature review and a desk review. In the methods section, the desk review IS a literature review. Also, it would be better to describe this step in terms of a convergent analysis across phases of the project (as was announced by the authors at the end of the methods), rather than collating evidence.

The final paragraph of the results which provides a more integrated analysis provides the richest portrait of the situation. It would be very interesting to have the authors expand on this analysis by fleshing out the insights generated by looking across the different components of their project and in relation to these main challenges.

Discussion

This section helpfully puts the study findings in context and offers a series of recommendations building up from the results. I suggest adding a table or figure that gathers up all the main recommendations.

This statement is not clear: “Furthermore a consistent prioritization technique was used that helped standardize inputs more representative.” The technique is not mentioned in the methods and it is not clear what it entails, nor what purpose it served.

Do the authors have reflections or recommendations regarded the methods they chose and how they were implemented? For example, reflections on the scope of the stakeholder mapping or including KIIs from other groups? Are there lessons that can be drawn for others who may wish to implement a similar process in another country (e.g. steps that could be added, obstacles to avoid)?

Overall:

The article is well written and has a clear structure. There are occasional grammatical errors and missing words in the text that should be attended to with a close copy edit.

**********

6. PLOS authors have the option to publish the peer review history of their article (what does this mean?). If published, this will include your full peer review and any attached files.

**Do you want your identity to be public for this peer review?**  If you choose “no”, your identity will remain anonymous but your review may still be made public.

For information about this choice, including consent withdrawal, please see our Privacy Policy.

Reviewer #1: No

Reviewer #2: No

**********

[NOTE: If reviewer comments were submitted as an attachment file, they will be attached to this email and accessible via the submission site. Please log into your account, locate the manuscript record, and check for the action link "View Attachments". If this link does not appear, there are no attachment files.]

-->Figure Resubmissions:

While revising your submission, we strongly recommend that you use PLOS’s NAAS tool (https://ngplosjournals.pagemajik.ai/artanalysis) to test your figure files. NAAS can convert your figure files to the TIFF file type and meet basic requirements (such as print size, resolution), or provide you with a report on issues that do not meet our requirements and that NAAS cannot fix.

After uploading your figures to PLOS’s NAAS tool - https://ngplosjournals.pagemajik.ai/artanalysis, NAAS will process the files provided and display the results in the "Uploaded Files" section of the page as the processing is complete. If the uploaded figures meet our requirements (or NAAS is able to fix the files to meet our requirements), the figure will be marked as "fixed" above. If NAAS is unable to fix the files, a red "failed" label will appear above. When NAAS has confirmed that the figure files meet our requirements, please download the file via the download option, and include these NAAS processed figure files when submitting your revised manuscript.

# Author comment: Author response to Decision Letter 1

**DOI:** 10.1371/journal.pgph.0005964.r002

25 Apr 2026

Attachment Submitted filename: Respons to Reviewers - 2026.04.25.docx

# Editor report: Decision Letter 1

**DOI:** 10.1371/journal.pgph.0005964.r003

**Authors:** Shashika Bandara

24 May 2026

PGPH-D-26-00295R1

Challenges in strengthening multi-sectoral action for optimum preparedness and response for public health emergencies in Sri Lanka

PLOS Global Public Health

Dear Dr. Arnold,

Thank you for submitting your manuscript to PLOS Global Public Health. After careful consideration, we feel that it has merit but does not fully meet PLOS Global Public Health’s publication criteria as it currently stands. Therefore, we invite you to submit a revised version of the manuscript that addresses the points raised during the review process.

Please submit your revised manuscript by Jun 23 2026 11:59PM. If you will need more time than this to complete your revisions, please reply to this message or contact the journal office at globalpubhealth@plos.org. When you're ready to submit your revision, log on to https://www.editorialmanager.com/pgph/ and select the 'Submissions Needing Revision' folder to locate your manuscript file.

Please include the following items when submitting your revised manuscript:

- A letter that responds to each point raised by the editor and reviewer(s). You should upload this letter as a separate file labeled 'Response to Reviewers'.
- A marked-up copy of your manuscript that highlights changes made to the original version. You should upload this as a separate file labeled 'Revised Manuscript with Track Changes'.
- An unmarked version of your revised paper without tracked changes. You should upload this as a separate file labeled 'Manuscript'.

Guidelines for resubmitting your figure files are available below the reviewer comments at the end of this letter.

As the corresponding author, your ORCID iD is verified in the submission system and will appear in the published article. PLOS supports the use of ORCID, and we encourage all coauthors to register for an ORCID iD and use it as well. Please encourage your coauthors to verify their ORCID iD within the submission system before final acceptance, as unverified ORCID iDs will not appear in the published article. *Only*  the individual author can complete the verification step; PLOS staff *cannot*  verify ORCID iDs on behalf of authors.

We look forward to receiving your revised manuscript.

Kind regards,

Shashika Bandara

Academic Editor

PLOS Global Public Health

Journal Requirements:

If the reviewer comments include a recommendation to cite specific previously published works, please review and evaluate these publications to determine whether they are relevant and should be cited. There is no requirement to cite these works unless the editor has indicated otherwise.

Please review your reference list to ensure that it is complete and correct. If you have cited papers that have been retracted, please include the rationale for doing so in the manuscript text, or remove these references and replace them with relevant current references. Any changes to the reference list should be mentioned in the rebuttal letter that accompanies your revised manuscript. If you need to cite a retracted article, indicate the article’s retracted status in the References list and also include a citation and full reference for the retraction notice.

Additional Editor Comments (if provided):

Dear Authorship team,

Thanks for taking the time to comprehensively revise the paper. The methods and approach is markedly clearer. However, there are some gaps in clarity and consistency of presentation of the paper. To make it easier for the authorship team, I have made comments in the tracked changes version of the paper (Revision 1) with suggested edits and relevant reframing. Where relevant I have suggested additional citations (two places). Since this paper will be read by a global audience, I have made edits to ensure a generalist, global audience can also understand the results presented. I believe these edits are relatively easy to make. If you have questions as you revise, please reach out to me - I will be happy to support with clarifications.

This paper as mentioned, will be an excellent addition to strengthen policy in Sri Lanka.

Thanks,

Shashika

[Note: HTML markup is below. Please do not edit.]

Reviewers' comments:

[NOTE: If reviewer comments were submitted as an attachment file, they will be attached to this email and accessible via the submission site. Please log into your account, locate the manuscript record, and check for the action link "View Attachments". If this link does not appear, there are no attachment files.]

Figure Resubmissions:

While revising your submission, we strongly recommend that you use PLOS’s NAAS tool (https://ngplosjournals.pagemajik.ai/artanalysis) to test your figure files. NAAS can convert your figure files to the TIFF file type and meet basic requirements (such as print size, resolution), or provide you with a report on issues that do not meet our requirements and that NAAS cannot fix.

After uploading your figures to PLOS’s NAAS tool - https://ngplosjournals.pagemajik.ai/artanalysis, NAAS will process the files provided and display the results in the "Uploaded Files" section of the page as the processing is complete. If the uploaded figures meet our requirements (or NAAS is able to fix the files to meet our requirements), the figure will be marked as "fixed" above. If NAAS is unable to fix the files, a red "failed" label will appear above. When NAAS has confirmed that the figure files meet our requirements, please download the file via the download option, and include these NAAS processed figure files when submitting your revised manuscript.

Attachment Submitted filename: PGPH-D-26-00295_R1_SLB.pdf

# Author comment: Author response to Decision Letter 2

**DOI:** 10.1371/journal.pgph.0005964.r004

28 May 2026

Attachment Submitted filename: Respons to reviewers -28.05.2026.docx

# Editor report: Decision Letter 2

**DOI:** 10.1371/journal.pgph.0005964.r005

**Authors:** Shashika Bandara

8 Jun 2026

Challenges in strengthening multi-sectoral action for optimum preparedness and response for public health emergencies in Sri Lanka

PGPH-D-26-00295R2

Dear Dr Arnold,

We are pleased to inform you that your manuscript 'Challenges in strengthening multi-sectoral action for optimum preparedness and response for public health emergencies in Sri Lanka' has been provisionally accepted for publication in PLOS Global Public Health.

Before your manuscript can be formally accepted you will need to complete some formatting changes, which you will receive in a follow up email. A member of our team will be in touch with a set of requests.

Please note that your manuscript will not be scheduled for publication until you have made the required changes, so a swift response is appreciated.

IMPORTANT: The editorial review process is now complete. PLOS will only permit corrections to spelling, formatting or significant scientific errors from this point onwards. Requests for major changes, or any which affect the scientific understanding of your work, will cause delays to the publication date of your manuscript.

If your institution or institutions have a press office, please notify them about your upcoming paper to help maximize its impact. If they'll be preparing press materials, please inform our press team as soon as possible -- no later than 48 hours after receiving the formal acceptance. Your manuscript will remain under strict press embargo until 2 pm Eastern Time on the date of publication. For more information, please contact globalpubhealth@plos.org.

Thank you again for supporting Open Access publishing; we are looking forward to publishing your work in PLOS Global Public Health.

Best regards,

Shashika Bandara

Academic Editor

PLOS Global Public Health

***********************************************************

Dear Dr. Arnold and team,

Thanks for your work on revising this paper. I see some typos remaining (for example, "within the and nine provinces" should be "within the nine provinces". I have accepted the paper - in proofing stage, please make sure you look for all typos and correct them.

Thanks,

Shashika

Reviewer Comments (if any, and for reference):
